# Utilizing genomic prediction to boost hybrid performance in a sweet corn breeding program

**DOI:** 10.3389/fpls.2024.1293307

**Published:** 2024-04-25

**Authors:** Marco Antônio Peixoto, Kristen A. Leach, Diego Jarquin, Patrick Flannery, Jared Zystro, William F. Tracy, Leonardo Bhering, Márcio F. R. Resende

**Affiliations:** ^1^ Laboratório de Biometria, Universidade Federal de Viçosa, Viçosa, Minas Gerais, Brazil; ^2^ Department of Horticultural Sciences, University of Florida, Gainesville, FL, United States; ^3^ Department of Agronomy, University of Florida, Gainesville, FL, United States; ^4^ Department of Plant and Agroecosystem Sciences, University of Wisconsin-Madison, Madison, WI, United States; ^5^ Organic Seed Alliance, Port Townsend, WA, United States

**Keywords:** G×E interaction, hybrid prediction, non-additive effects, RKHS model, cross-validation schemes

## Abstract

Sweet corn breeding programs, like field corn, focus on the development of elite inbred lines to produce commercial hybrids. For this reason, genomic selection models can help the *in silico* prediction of hybrid crosses from the elite lines, which is hypothesized to improve the test cross scheme, leading to higher genetic gain in a breeding program. This study aimed to explore the potential of implementing genomic selection in a sweet corn breeding program through hybrid prediction in a within-site across-year and across-site framework. A total of 506 hybrids were evaluated in six environments (California, Florida, and Wisconsin, in the years 2020 and 2021). A total of 20 traits from three different groups were measured (plant-, ear-, and flavor-related traits) across the six environments. Eight statistical models were considered for prediction, as the combination of two genomic prediction models (GBLUP and RKHS) with two different kernels (additive and additive + dominance), and in a single- and multi-trait framework. Also, three different cross-validation schemes were tested (CV1, CV0, and CV00). The different models were then compared based on the correlation between the estimated breeding values/total genetic values and phenotypic measurements. Overall, heritabilities and correlations varied among the traits. The models implemented showed good accuracies for trait prediction. The GBLUP implementation outperformed RKHS in all cross-validation schemes and models. Models with additive plus dominance kernels presented a slight improvement over the models with only additive kernels for some of the models examined. In addition, models for within-site across-year and across-site performed better in the CV0 than the CV00 scheme, on average. Hence, GBLUP should be considered as a standard model for sweet corn hybrid prediction. In addition, we found that the implementation of genomic prediction in a sweet corn breeding program presented reliable results, which can improve the testcross stage by identifying the top candidates that will reach advanced field-testing stages.

## 1 Introduction

Hybrid breeding programs are the standard approach for the development of commercial products for multiple crops. Hybrid plants can be generated through a simple cross between individuals that may or may not belong to different genetic groups, such as heterotic groups (Labroo et al., 2021). A definition of heterotic groups here assumed is a group of individuals/genotypes from a population (or different populations) that presents similar performance in terms of combining ability in addition to heterotic response when they are crossed with individuals/genotypes from other genetically distinct groups (Melchinger and Gumber, 1998; Labroo et al., 2021). In hybrid crop production, parents are typically evaluated based on hybrid performance resulting from a cross with known elite inbred testers. Hybrids that perform well are then evaluated in an increasing number of representative environments or target populations of environments and against additional testers. Selection of the best inbreds, depending on the stage of product development, can be performed using the general combining ability (GCA), which represents how good is a parent, based on the hybrid performance for all crosses that it was part of, or a combination of GCA and the specific combining ability, indicating how superior the hybrid is compared to the mean of its parents.

One of the challenges in hybrid breeding programs is the testing of all the possible crosses among elite inbreds as this is generally not practical. For example, in a program with 100 potential parents (*N*), the number of possible crosses is 4,950 $\left(C=\frac{N*(N-1)}{2}\right)$ , assuming no reciprocal crosses. Similarly, during the testing stage, the number of putative crosses that can be generated among a set of lines and the elite testers can become quite large (Zystro et al., 2021b). A modern hybrid breeding program can be divided into two phases: line development followed by product development, where hybrids are created and tested (Cowling et al., 2020; Powell et al., 2020). Genomic selection can aid in both phases, but particularly for product development. In this approach, parental genotypes are utilized to predict hybrid performance, which is expected to increase the probability of superior hybrids in field-testing stages (Marinho et al., 2022).

Sweet corn is a vegetable crop and hybrids are developed to address producer and consumer needs. While hybrid vigor is present, the presence of heterotic groups is not as well defined as in field corn. This imposes challenges in the implementation of reciprocal recurrent selection in the breeding program (Peixoto et al., 2023). A second challenge in sweet corn breeding compared to field corn is a demand for consumer-oriented traits, in addition to yield. In response, the breeder must account for all these demands before releasing a hybrid.

Genomic hybrid prediction is applied broadly in maize breeding (Fritsche-Neto et al., 2021). Similarly, statistical methods have been evolving and developed for genomic predictions. Generally, the genomic models used for hybrid prediction are genomic BLUP (GBLUP) or reproducing kernel Hilbert space (RKHS) (Alves et al., 2019; Cuevas et al., 2019; Krause et al., 2020; Marinho et al., 2022). Even though similar in implementation, the differences between GBLUP and RKHS are related to the mathematical equations that are used to build the genetic relationship matrix (or kernels) which are then used in the models for prediction (VanRaden, 2008; de los Campos et al., 2010; Vitezica et al., 2013). In addition, methods that consider both additive and non-additive effects have demonstrated increases in trait predictability (Ferrão et al., 2020; Oliveira et al., 2020).

When genomic selection is applied to a situation where several traits are important for selection, models can leverage the correlation between traits (Covarrubias-Pazaran et al., 2018) increasing the prediction accuracy for traits with lower heritabilities. This happens by borrowing information from traits with higher heritability (Mrode, 2014). Lastly, the deployment of genomic prediction needs to accommodate a plan to deal with genotype-by-environment interaction (G×E). As demonstrated by Jarquín et al. (2014) the G×E interaction impacts the genotype ranking making it difficult to calibrate the model across multiple environments. In this study, we explore the use of genomic selection in sweet corn breeding and evaluate different statistical models to account for multiple traits and multiple environments.

## 2 Materials and methods

### 2.1 Plant material

A total of 506 hybrids were created from 62 lines that came from a collaboration between the University of Florida and the University of Wisconsin’s sweet corn breeding programs. The hybrids were evaluated in three sites: Florida (FL), California (CA), and Wisconsin (WI) across two years, 2020 and 2021. Here, we deemed an environment to be the combination of sites and years (six environments in total) (**Supplementary material – Supplementary Figure S1**). In the CA location, 246 of the hybrids were measured in the 2020 season (CA20) and 39 hybrids in the 2021 season (CA21). In the FL location, we evaluated 418 and 203 hybrids in the 2020 (FL20) and 2021 (FL21) seasons, respectively. For the WI site, 236 and 39 hybrids were assessed, in the 2020 and 2021 seasons (WI20 and WI21), respectively.

### 2.2 Phenotyping

Hybrids in FL20, FL21, and CA20 were planted following an augmented randomized incomplete block design, with two replications and 10 blocks (FL20, CA20) and six blocks (FL21). The experiments in CA21, WI20, and WI21, were planted in a randomized complete block design with three replications (CA21 and WI21) and two replications (WI20). A total of 20 traits were measured over the six environments. The traits can be grouped into three different categories: Plant-related traits (STC: stand count; DTP: days to pollination; DTS: days to silking; PH: plant height; EH: ear height), ear traits (EL: ear length. EW: ear width; TPF: tip fill; HP: husk protection; KRN: kernel row number; SOL: solidity; TP: taper; CUR: ear curvature; HAP: husk appearance; RAP: row appearance; ES: ear shape; CR: color rate; RT: over-all ear rating), and flavor-related traits (FLA: flavor; TXT: texture). Not all traits were measured in all localities, being only the traits EL, EW, and TPF being measured in all six environments. A thorough list of all the traits and localities where they were or were not measured is presented in the **Supplementary material – Supplementary Table S1**.

In addition, a scheme of the crossing block used to generate the hybrids is represented (**Supplementary material – Supplementary Figure S1**), showing the contribution of each line (parent) for the hybrid production. Elite lines from the University of Florida were crossed against the elite lines from the University of Wisconsin. The ear traits measured in the FL site were measured with the auxiliary of EarCV (Gonzalez et al., 2022), whilst flavor, texture, rating, tip fill, husk protection, row appearance, color rate, and ear shape at the WI site were rated at the fresh eating stage on 1 to 5 scale, with 5 as the best for all traits.

### 2.3 Phenotypic analyses

In the first step of the analysis, the estimation of variance components and prediction of genotypic values for the traits assessed was made via the residual maximum likelihood/best linear unbiased prediction (REML/BLUP) procedure. The statistical model used for the analyses of the data from CA21, WI20, and WI21, evaluation of hybrids in a randomized complete block design with one observation per plot, was given by the following equation:


y=Xr+Zg+e,

where *y* is the vector of phenotypes; *r* is the vector of replication effects (assumed as fixed), added to the overall mean; *g* is the vector genotypic effects [(assumed as random) $g∼N(0,\sigma_{g}^{2})$ , where $\sigma_{g}^{2}$ is the genotypic variance]; and *e* is the vector of residuals [(random) $e∼N(0,\sigma_{e}^{2})$ , where $\sigma_{e}^{2}$ is the residual variance]. Uppercase letters (*X* and *Z*) represent the incidence matrices for *r* and *g*, respectively.

For FL20, FL21, and CA20 environments, the experimental design used for the evaluation of hybrids was an augmented randomized incomplete block design with one observation per plot. The statistical model is given by the following equation:


y=Xr+Zg+Wp+e,

where *r* is the vector of checks inside blocks and replication effects (both assumed as fixed), added to the overall mean; *p* is the vector block effects [(assumed as random) $p∼N(0,\sigma_{p}^{2})$ , where $\sigma_{p}^{2}$ is the block variance] and *W* represents the incidence matrix to *p*.

For the random effects, significance was tested by the likelihood ratio test (LRT) using a chi-square statistic with 1 degree of freedom and a 5% probability of type I error (Rao, 1952). To access the linear relationship between pairs of traits, correlations were determined between paired vectors of trait BLUPs, in each environment. We used BLUPs to estimate these correlations due to the unbalanced experimental design implemented in some environments. Best linear unbiased estimation (BLUE) values were generated using the same equation above, assuming genotypes as a fixed effect. The BLUEs were promptly used in the second stage (see topic 2.5.1 below), which avoids double penalization in the genetic effects that may be caused by shrinking the effects twice (phenotypic BLUPs followed by GBLUP).

### 2.4 Genotyping

For each line considered as a parent, DNA was extracted and sequenced with NovaSeq 2x150bp reads. The average depth was 8.45x (4.2x to 14.5x). Whole genome sequences were aligned to that Ia453-*sh2* genome (Hu et al., 2021) using BWA-MEM. From the total of variants called (Colantonio, 2023), a subset of 200,000 SNP markers was randomly sampled using vcftools (Danecek et al., 2011). The SNPs with a minor allele frequency of less than 1% and missing data of greater than 30% were removed from the SNP data set. After filtering, a final set of 37,229 SNPs was used for the analyses.

### 2.5 Genomic selection models

The GBLUP (VanRaden, 2008) and RKHS (de los Campos et al., 2009) models were evaluated within a framework including both single- and multi-traits. The models considered additive effects (A) or additive plus dominance effects (AD), totalizing eight models, as follows: single trait GBLUP with additive effect (AG) and additive plus dominance effect (ADG), multi trait GBLUP with additive effect (MAG) and additive plus dominance effect (MADG), single trait RKHS with additive effect (AR) and additive plus dominance effect (ADR), multi trait RKHS with additive (MAR) and additive plus dominance effect (MADR).

**Figure 1:**
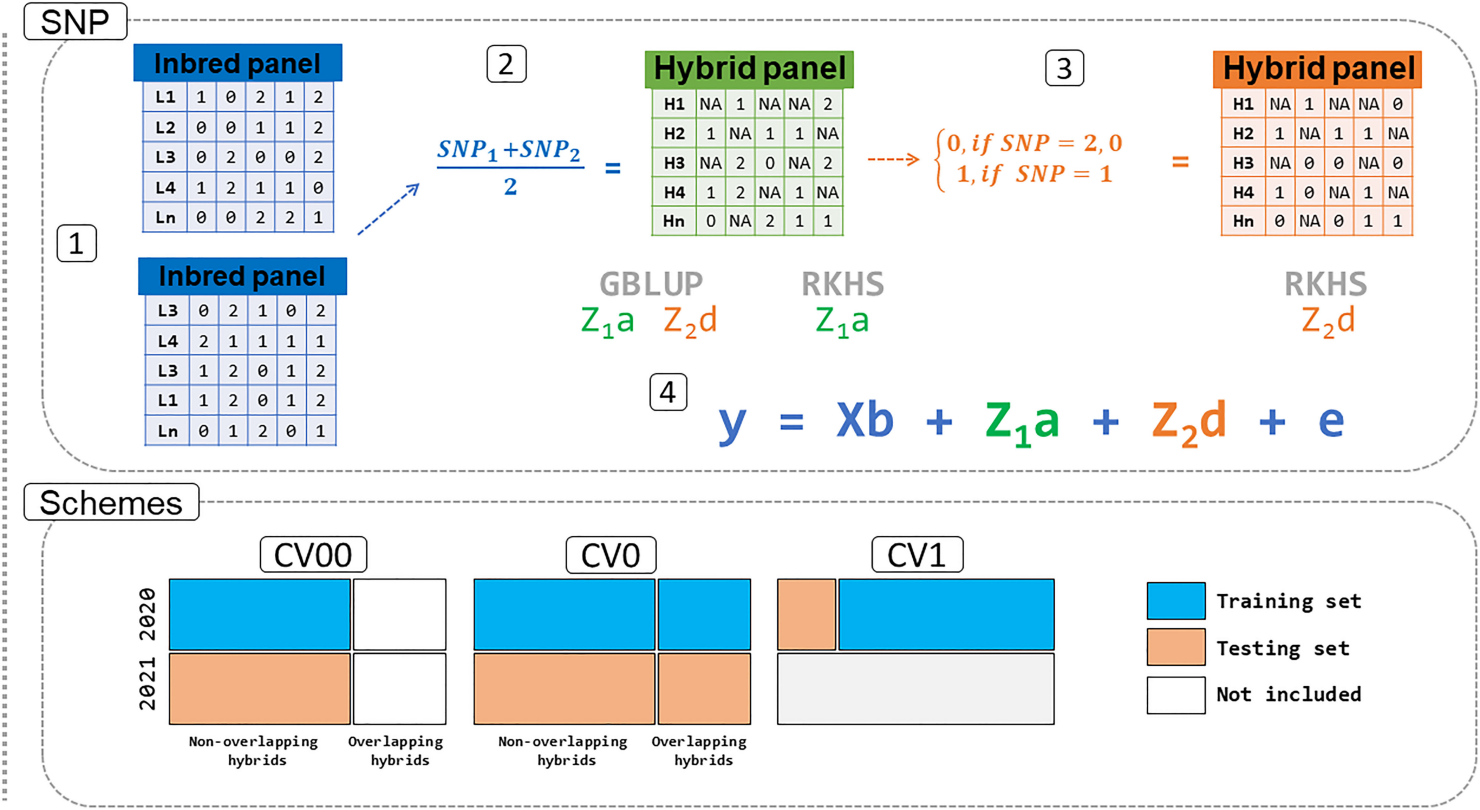
Pipeline for the prediction in the sweet corn dataset. The SNP panel for lines (1) was combined based on the formula in (2) and coded to the additive (*Z*_1_ *a*) and dominance (*Z*_1_ *d*) kernels. One additional step is needed to implement *Z*_1_ *d* for the RKHS model (3). All information generated is then used in step (4) for the prediction of genomic estimated breeding values (GEBV) using GBLUP and RKHS models. The schemes tested in this study are CV00: untested hybrids in untested environments. CV0: tested hybrids in untested environments. CV1: untested hybrids in tested environments. SNP: single nucleotide polymorphism. GBLUP: genomic best linear unbiased prediction. RKHS: reproducing kernel Hilbert space.

For the dominance matrices, the SNPs matrix was coded as 0 for both homozygous classes (AA and aa) and as 1 for the heterozygous class, whereas the intermediated values (0.5 and 1.5) that came from one homozygous locus and one heterozygous locus from a pair of inbreds were coded as missing data (**Figure 1**). We describe the model’s structure below.

#### 2.5.1 Genomic best linear unbiased prediction

The GBLUP model for single- and multi-trait GBLUP model with additive effects and additive plus dominance effects are given by:


y=μ+Za+e,

$$y=\mu +Z_{1}a+Z_{2}d+e,$$

where *y* is the vector with BLUE values of all traits predicted from the hybrids in the first stage of the analyses, *μ* is the overall mean, *a* represents a vector of additive genetic effects for all traits, where $a∼N(0,\sum_{a}⊗G_{a})$ , *G_a_* is the additive genomic relationship matrix and Σ*_a_* is the additive genetic variance-covariance matrix across traits, *d* represents the vector of dominance effects, where $d∼N(0,\sum_{d}⊗G_{d})$ , where *G_d_* is the dominance genomic relationship matrix and Σ*_d_* is the dominance variance-covariance matrix across traits and ***e*** is the residual error variance, where $e∼N(0,\sum_{res}⊗I)$ , where Σ*_res_* is the residual variance-covariance matrix for all traits and I represents an incidence matrix. *Z*_1_ and *Z*_2_ represent the incidence matrices to *a* and *d* effects, respectively. It should be noted that for the single trait analyses, the covariance matrices used in the multi-trait model (Σ*_a_*, Σ*_d_*, Σ*_res_*) become scalar values, representing the variance for the trait. In this case, $a∼N(0,G_{a}\sigma_{a}^{2})$ , where $\sigma_{a}^{2}$ is the additive genetic variance, $d∼N(0,G_{d}\sigma_{d}^{2})$ , where is the dominance variance, and $e∼N(0,I\sigma_{e}^{2})$ , where $\sigma_{e}^{2}$ is the residual variance.

The additive and dominance relationship matrices for the GBLUP-based model were constructed using AGHmatrix (Amadeu et al., 2016, 2023), following the parametrization of VanRaden (2008) and Vitezica et al. (2013). The missing markers’ values were replaced by the markers’ mean, default in the package AGHmatrix.

#### 2.5.2 Reproducing Kernel Hilbert space

A model, consisting of the semi-parametric kernel RKHS, was used for single- and multi-trait prediction, using additive and additive + dominance effects models. The RKHS model includes both, additive and non-additive gene effects implicitly, such as epistatic effects (Gianola and Van Kaam, 2008; Jiang and Reif, 2015; Almeida-Filho et al., 2019). The following models were used:


y=μ+Za+e,

$$y=\mu +Z_{1}a+Z_{2}d+e,$$

where *y* is the vector of BLUE values from the hybrids predicted in the first stage of the analyses, μ is the overall mean, *a* represents the vector of additive and additive-additive epistatic genetic effects, where $a∼N(0,\sum_{a}⊗K_{a})$ , where *K_a_* is the additive symmetric semipositive definite matrices representing the covariance of the genetic values and Σ*_a_* is the additive variance-covariance matrix across traits, *d* represents a vector of dominance and dominance-dominance epistatic effects, where $a∼N(0,\sum_{d}⊗K_{d})$ , where *K_d_* is the dominance symmetric semipositive definite matrices representing the covariance of the genetic values and Σ*_d_* is the dominance variance-covariance matrix across traits, and *e* is the residual error variance, where $e∼N(0,\sum_{res}⊗I)$ , where Σ*_res_* is the residual variance-covariance matrix for all traits. *Z*_1_ and *Z*_2_ represent the incidence matrices to *a* and *d* effects, respectively. The previously multi-trait model notation above described holds for the single-trait model. Here, for the additive component $a∼N(0,K_{a}\sigma_{a}^{2})$ , for the dominance component $d∼N(0,G_{d}\sigma_{d}^{2})$ , where $\sigma_{d}^{2}$ is the dominance variance, and the residuals follows $e∼N(0,I\sigma_{e}^{2})$ .

In the RKHS, *K_a_* and *K_d_* are represented by: $K_{a}=\text{exp}\left(-\varphi_{a_{r}}D_{a}^{2}\right)$ , and $K_{d}=\text{exp}\left(-\varphi_{d_{r}}D_{d}^{2}\right)$ , where $\varphi_{a_{r}}$ and $\varphi_{d_{r}}$ represents the bandwidth parameters for additive and dominance models (Pérez-Rodríguez and de los Campos, 2014) and $D_{a}^{2}$ and $D_{d}^{2}$ represents the Euclidian distance matrix using the SNP matrix for additive effects, and the dominance matrix for the additive effects, respectively. Here, we followed Morota and Gianola (2014) and applied the kernel averaging model, where *K_a_* and *K_d_* were represented for three kernels that came from the three different values of the bandwidth parameters ( $\varphi_{a_{r}}$ and $\varphi_{d_{r}}$ ), represented by 5/h, 1/h and 0.2/h, where h is the 5^th^ percentile of the $D_{a}^{2}$ or $D_{d}^{2}$ leading to local, intermediate and global kernels, respectively (Morota and Gianola, 2014).

The additive and dominance RKHS kernels were built based on a custom function available together with the main code. Following the parametrization mentioned for the GBLUP model, the missing values were replaced by the markers’ mean.

#### 2.5.3 Models under genotype-by-environment interaction

We expand the models before mentioned by including an interaction term between genotypes and environments (Jarquín et al., 2014). The models are as follows:


$$y=\mu +Z_{E}\beta +Z_{1}a+u_{AE}+e,$$

$$y=\mu +Z_{E}\beta +Z_{1}a+Z_{2}d+u_{AE}+u_{DE}+e,$$

where *Z_E_* represents the incidence matrix for the effects of environments (*i*.*e*., a matrix that connects the phenotypes with environments), *β* is the fixed effect of environment, and for the RKHS model: $u_{AE}\;\sim \;N\left(0,\Sigma_{AE}⊗K_{AE}\right)$ and $u_{DE}\;\sim \;N\left(0,\Sigma_{DE}⊗K_{DE}\right)$ , being $K_{AE}=\;Z_{E}{\mathrm{Z'}}_{E}⊙\;Z_{1}K_{a}{\mathrm{Z'}}_{1}\;$ and $K_{DE}=\;Z_{E}{\mathrm{Z'}}_{E}⊙\;Z_{1}K_{d}{\mathrm{Z'}}_{1}$ , Σ*_AE_* and Σ*_DE_* is the variance-covariance matrix for AE and DE interaction effects across traits, and for the GBLUP model: $u_{AE}\;\sim \;N\left(0,\Sigma_{AE}⊗G_{AE}\right)$ and $u_{DE}\;\sim \;N\left(0,\Sigma_{DE}⊗G_{DE}\right)$ , $G_{AE}=\;Z_{E}{\mathrm{Z'}}_{E}⊙\;Z_{1}G_{a}{\mathrm{Z'}}_{1}\;$ and $G_{AD}=\;Z_{E}{\mathrm{Z'}}_{E}⊙\;Z_{1}G_{d}{\mathrm{Z'}}_{E}$ , being ⊙ the Hadamard product (Jarquín et al., 2014). This model accounts for the main effects of genotypes, the main effects of environments, and the interactions between genotypes and environments (Jarquín et al., 2014; Costa-Neto et al., 2021; Persa et al., 2023).

### 2.6 Cross-validation schemes

Three different cross-validation schemes were implemented in the analyses aiming to infer the models’ predictability. The first validation examined was within-environment cross-validation (untested hybrids in tested environments or CV1) for the three individual environments (CA20, FL20, and WI20, **Figure 1**). Five-fold cross-validation was used (20% of the hybrids as a testing set and 80% for training), and permutations in between those groups were implemented. In addition, the process was repeated 20 times. The models used were the ones described in sections 2.5.1-2.5.2, totaling eight model structures. A total of eight traits were used in the prediction for the California site (CA20) and for the Florida site (FL20) and 16 traits were used in the prediction for the Wisconsin site (WI20) (**Supplementary material, Supplementary Table S1**).

The second cross-validation scheme included tested hybrids in untested environments or CV0 (using the models described in section 2.5.1-2.5.2). We used hybrid data for each site in 2020 to train the model and then predict hybrids at each site in 2021, separately (*i*.*e*., CA20, FL20, and WI20 as a training set for the prediction of CA21, FL21, and WI21, respectively). As not all traits overlapped from 2020 with 2021, a total of eight, seven, and 14 traits were used in the model for the prediction of CA, FL, and WI sites, respectively (**Supplementary material, Supplementary Table S1**).

The third cross-validation was the untested hybrids in the untested environment or CV00 (using the models described in section 2.5.1-2.5.2), where we removed hybrid information that overlapped between years (**Figure 1**). Additionally, we used the same principles as before (CV0) to predict the information of hybrids in each site in 2020 as a training population to predict the performance of the hybrids in the subsequent year (2021). For the same reason, the same number of traits from CV0 were used here for the hybrid genomic prediction (eight, seven, and 14 traits for CA, FL, and WI, respectively).

In addition, we used the information of more than one site from 2020 (CA20, FL20, WI20) to predict the year 2021 (CA21, FL21, or WI21) (*e*.*g*., we calibrated the model using CA20, FL20, and WI20 data and predict CA21. We did all possible combinations in this case). The models implemented for this prediction were the ones described in the section 2.5.3. They account for the main effects of genotypes, environments, and the interactions between them. In this specific case, we only used the information of the traits EL, EW, and TPF (the only traits that were measured in all environments) to build the CV0 and CV00 schemes.

The model accuracy was calculated between the estimated breeding value and the BLUE values estimated in the first stage for each trait.

### 2.7 Computational implementation

The first analytic stage (phenotypic analyses) was carried out in the R package ASREML (Gilmour et al., 2015), with the genomic models being implemented in the BGLR package (Pérez-Rodríguez and de los Campos, 2014, 2022). The Bayesian models used 30,000 iterations, with a burn-in of 3000, and a sampling interval (thinning) of 10, totaling 2700 iterations. The models from the second stage were implemented in the Bayesian framework. For such, flat priors were considered for the intercept. In addition, the covariance matrices (for multi-trait models) and variance components (for single-trait models) were assumed to be unknown with distributions and hyperparameters defined using the default values in BGLR (for more details, please see Pérez-Rodríguez and de los Campos, 2014, 2022). In addition, all codes and data used for the analyses are available at: https://github.com/Resende-Lab/Peixoto_Sweet_corn_hybrid_prediction).

## 3 Results

Here, we implemented genomic prediction models for the prediction of hybrid performance accounting for several traits in a sweet corn breeding program. The results demonstrated that implementation is feasible, even though a large amount of variation was found in the phenotypic datasets. This contributed to dissimilarity patterns being observed for trait heritabilities and correlations between traits leading to variation in prediction accuracy for the traits. Overall, the GBLUP models outperformed the RKHS models in all cross-validation schemes. In addition, models accounting for additive plus dominance kernels presented a slight improvement for some traits and models.

### 3.1 Trait heritability, significance, and correlations

The LRT indicated that the genotypic effect was significant for all traits, except for CUR and SOL (FL21), and FLA (WI20) (**Supplementary material – Supplementary Table S2**). In addition, the block effect was not significant for most traits, except EW, TP, and TPF (FL20), EH, EW, and PH (CA20) (**Supplementary material – Supplementary Table S3**). Trait heritabilities varied across environments. For plant-related traits, the heritabilities varied from 0.28 (EH in CA21) to 0.82 (DTP in WI20). The ear-related traits presented heritabilities that varied from 0.12 (CUR in FL21) to 0.80 (EL in CA20). Whereas, FLA and TXT had heritabilities of 0.05 and 0.31, respectively. A summary of trait information for all six environments can be found in **Supplementary material – Supplementary Tables S1–S6**.

The correlations between paired vectors of trait BLUPs also demonstrated a wide range of variation (**Supplementary material – Supplementary Figures S2–S7**). From the total number of paired correlations for all traits in the six environments, 151 out of 316 (47.8%) were significant at 10%, representing the presence of a significant linear association with each other. Large correlation values were found between traits from the same group. For instance, days to pollination and days to silking were highly correlated (0.93 for WI20 and 0.94 for WI21), whereas plant height and ear height had correlations of 0.72 (WI20) and 0.86 (CA21). In addition, texture, and flavor (flavor-related traits) presented moderated values of correlation (0.25 in CA20 and 0.31 in CA21). Some negative correlation values were also observed, such as tip fill and ear length in CA20 (-0.53) and days to pollination and stand count (-0.6 in CA21).

### 3.2 Within-year hybrid prediction accuracy (CV1)

**Figure 2:**
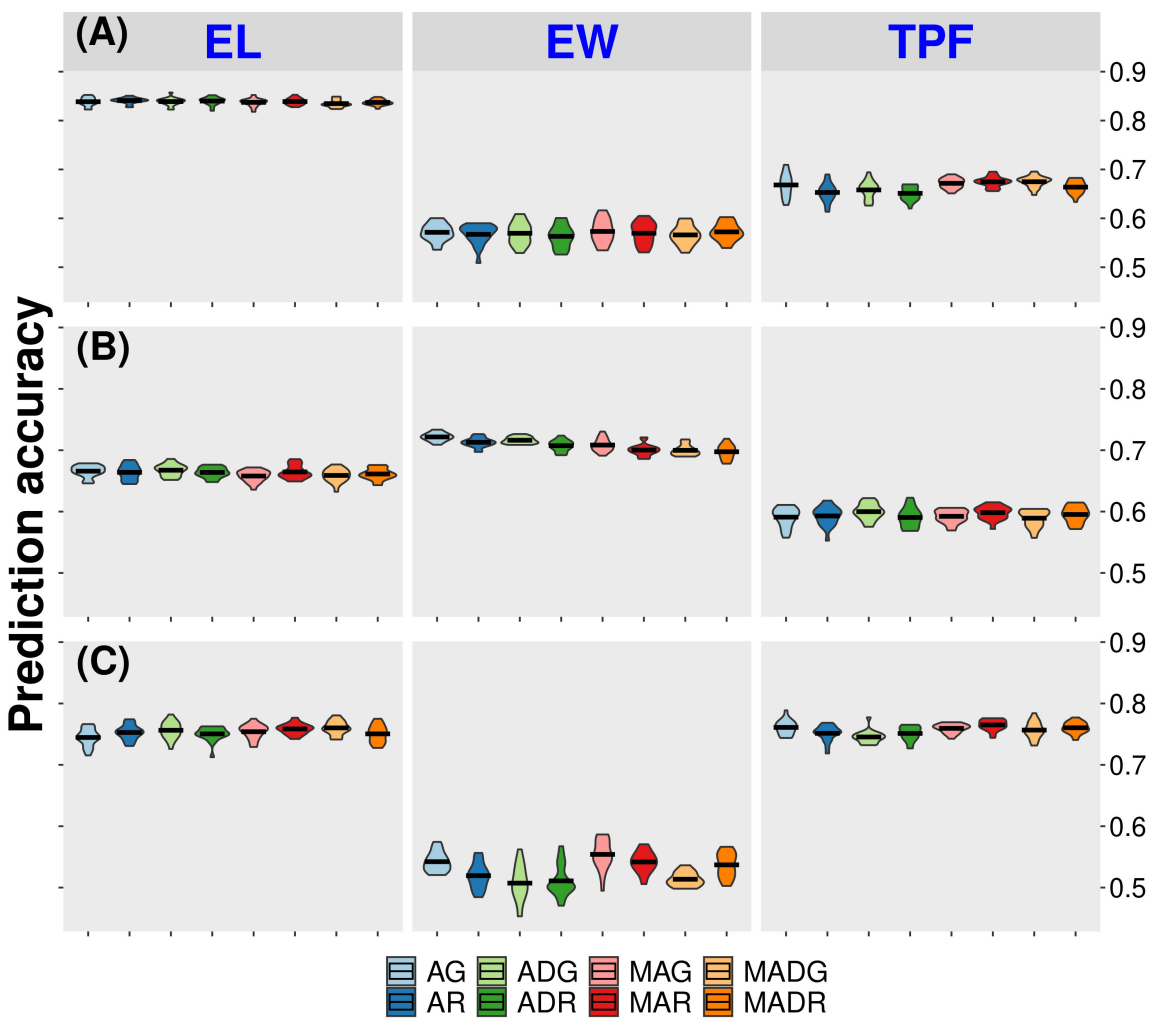
Accuracy for prediction of untested hybrids in tested environments (CV1). AG, GBLUP single-trait with additive effect; ADG, GBLUP single-trait with additive effect and dominance effect; MAG, GBLUP multi-trait with additive effect; MADG, GBLUP multi-trait with additive effect and dominance effect; AR, RKHS single-trait with additive effect; ADR, RKHS single-trait with additive effect and dominance effect; MAR, RKHS multi-trait with additive effect; MADR, RKHS multi-trait with additive effect and dominance effect. **(A)** California site. **(B)** Florida site. **(C)** Wisconsin site. EL = ear length. EW, ear width; TPF, tip fill.

We compared the prediction of all models using the CV1 approach (**Figure 2** and **Table 1**). In the CV1 approach, the model calibration and model validation take place in the same environment and within each given year. The models were then tested within each site, for all traits for the year of 2020 (CA20, FL20, and WI20), which had larger population sizes. Results show that RKHS was slightly overperformed by GBLUP. For the trait days to pollination, it improved from 0.449 to 0.465 (AR and AG models, respectively) and from 0.569 to 0.573 (MAR and MAG, respectively) for the trait ear width in the California site. In the Florida site, ear width prediction accuracy improved from 0.70 to 0.72 (AR and AG models, respectively) and the trait tip fill improved from 0.59 (ADR model) to 0.60 (ADG model). The model accuracies in the Wisconsin site for the GBLUP-based models were higher for traits as days to pollination and days to silking, improving from 0.835 and 0.834, respectively (AR model) to 0.837 (both traits under AG model) (**Supplementary material - Supplementary Figure S8–S10**).

**Table 1:** Prediction accuracy of within-site across-year hybrids prediction for California, Florida, and Wisconsin.

Trait	Model								Mean
	AG	ADG	MAG	MADG	AR	ADR	MAR	MADR	
California									0.662
DTP	0.465	0.440	0.465	0.460	0.449	0.451	0.465	**0.471**	0.458
EH	**0.832**	0.828	0.830	0.825	0.827	0.830	0.826	0.823	0.828
EL	0.839	0.839	0.837	0.835	**0.841**	0.840	0.839	0.837	0.838
EW	0.571	0.569	**0.573**	0.566	0.567	0.563	0.569	0.572	0.569
HP	0.730	0.731	0.729	0.734	**0.740**	0.736	0.738	0.739	0.735
PH	0.830	0.830	0.828	0.828	0.830	**0.836**	0.831	0.826	0.830
STC	**0.393**	0.360	0.390	0.372	0.357	0.337	0.379	0.378	0.371
TPF	0.668	0.658	0.672	**0.675**	0.653	0.651	0.675	0.664	0.664
Florida									0.546
CUR	0.357	0.345	**0.360**	0.321	0.343	0.340	0.347	0.352	0.346
EL	0.666	**0.667**	0.658	0.659	0.664	0.664	0.665	0.661	0.663
EW	**0.722**	0.716	0.708	0.700	0.713	0.707	0.700	0.697	0.708
KRN	0.765	0.764	0.757	0.761	0.762	**0.767**	0.762	0.761	0.762
SOL	0.444	0.445	0.440	0.426	0.450	0.449	0.447	**0.457**	0.444
STC	**0.311**	0.308	0.310	0.294	0.294	0.285	0.293	0.285	0.298
TP	0.550	0.558	0.556	**0.565**	0.552	0.552	0.559	0.563	0.557
TPF	0.591	**0.600**	0.592	0.589	0.593	0.590	0.598	0.595	0.594
Wisconsin									0.621
CR	0.490	0.481	0.477	0.469	0.483	0.475	**0.499**	0.489	0.483
DTP	0.830	**0.837**	0.833	0.837	0.835	0.832	0.834	0.832	0.834
DTS	0.832	**0.837**	0.832	0.834	0.834	0.832	0.833	0.827	0.833
EH	0.711	0.711	0.722	**0.724**	0.703	0.705	0.716	0.715	0.713
EL	0.745	0.756	0.754	**0.760**	0.753	0.750	0.758	0.750	0.753
ES	0.541	0.521	0.541	0.523	0.544	0.541	**0.552**	0.544	0.539
EW	0.542	0.507	**0.554**	0.514	0.519	0.511	0.542	0.537	0.528
FLA	0.271	0.223	**0.297**	0.269	0.279	0.269	**0.297**	0.286	0.274
HAP	0.465	0.457	0.463	0.460	**0.479**	0.466	0.475	0.461	0.466
HP	**0.722**	0.715	0.713	0.709	0.719	0.715	0.717	0.711	0.715
KRN	0.790	0.789	0.790	0.778	0.786	0.789	**0.792**	0.780	0.787
PH	0.817	0.816	0.819	0.810	0.816	**0.821**	0.814	0.810	0.815
RAP	0.446	0.448	0.456	0.450	0.472	0.470	**0.480**	0.473	0.462
RT	0.537	0.518	**0.547**	0.501	0.510	0.523	0.539	0.535	0.526
TPF	0.761	0.745	0.759	0.757	0.751	0.751	**0.765**	0.760	0.756
TXT	0.463	0.427	**0.487**	0.433	0.453	0.446	0.479	0.464	0.456

CR, color rate; CUR, curvature; DTP, days to pollination; DTS, days to silking; EL, ear length; EH, ear height; ES, ear shape; EW, ear width; FLA, flavor; HAP, husk appearance; HP, husk protection; KRN, kernel row number; PH, plant height; RAP, Row appearance; RT, rating; SOL, solidity; STC, stand count; TP, taper; TPF, tip fill; TXT, texture; AG, GBLUP single-trait with additive effect; ADG, GBLUP single-trait with additive effect and dominance effect; MAG, GBLUP multi-trait with additive effect; MADG, GBLUP multi-trait with additive effect and dominance effect; AR, RKHS single-trait with additive effect; ADR, RKHS single-trait with additive effect and dominance effect; MAR, RKHS multi-trait with additive effect; MADR, RKHS multi-trait with additive effect and dominance effect.

CV1= Prediction of untested hybrids in tested environments. Bolded numbers represent the model with the highest performance for each trait.

On the other hand, models accounting for multi-traits improved trait predictability by 0.76% for CA20 and 0.89% for WI20, whereas models with single traits were better in FL20 (0.55% higher). The inclusion of dominance in the model (AD models) was outperformed by models with only additive effects (0.63% at CA20, 0.47% at FL21, 1.40% at WI20). In addition, prediction accuracy was higher than 0.8 for some traits, such as EL, EH, and PH in CA20 and DTP, DTS, and PH in WI20. Additionally, the STC trait had lower predictability in both CA20 and FL20, with values of 0.371 and 0.298, respectively, and the flavor trait presented a lower value in WI20 (0.274).

### 3.3 Within-site across-year hybrid prediction accuracy (CV0)

**Figure 3:**
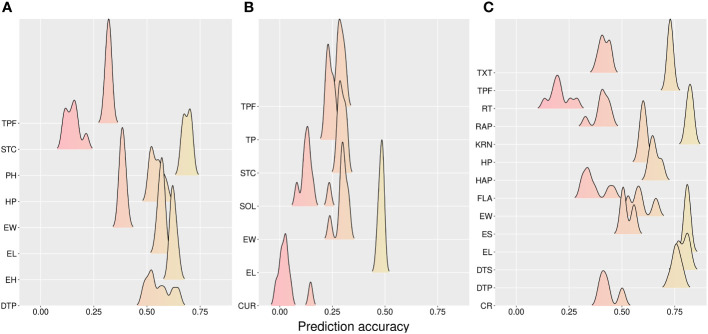
Accuracy for prediction across methods for tested hybrids in new environments (CV0). **(A)** California, **(B)** Florida, and **(C)** Wisconsin sites. CR, color rate; CUR, curvature; DTP, days to pollination; DTS, days to silking; EL, ear length; EH, ear height; ES, ear shape; EW, ear width; FLA, flavor; HAP, husk appearance; HP, husk protection; KRN, kernel row number; PH, plant height; RAP, row appearance; RT, rating; SOL, solidity; STC, stand count; TP, taper; TPF, tip fill; TXT, texture.

Single-trait models were better than multi-trait models for the CV0 scheme, *e*.*g*., 2.84% and 2.3% for CA and WI, respectively (**Table 2**). Additive models had higher prediction accuracies than AD models alone (3.79%, 4.45%, and 3.28% improvement at CA, FL, and WI, respectively). The average prediction across models was weaker in FL (0.146 for CUR to 0.49 for EL) (**Figure 3**). In addition, higher accuracies were found in WI for the traits KRN (0.83) and EL (0.82). On average, the predictions were 0.479 (CA), 0.253 (FL), and 0.579 (WI).

**Table 2:** Prediction accuracy of within-site across-year hybrids prediction for California, Florida, and Wisconsin.

Trait	Model								Mean
	AG	ADG	MAG	MADG	AR	ADR	MAR	MADR	
California									0.479
DTP	**0.648**	0.622	0.559	0.502	0.583	0.527	0.521	0.484	0.556
EH	0.640	0.621	**0.646**	0.626	0.622	0.611	0.619	0.613	0.625
EL	0.547	0.565	0.555	0.575	0.567	0.570	0.578	**0.581**	0.567
EW	0.393	0.373	**0.403**	0.379	0.385	0.381	0.394	0.378	0.386
HP	0.573	0.532	**0.592**	0.552	0.521	0.511	0.555	0.518	0.544
PH	0.707	**0.709**	0.681	0.674	0.702	0.702	0.666	0.670	0.689
STC	**0.214**	0.163	0.172	0.110	0.156	0.144	0.128	0.112	0.150
TPF	0.323	0.306	0.322	0.310	**0.329**	0.310	0.326	0.326	0.319
Florida									0.253
CUR	0.030	0.022	**0.146**	0.031	0.005	0.008	0.050	-0.017	0.034
EL	0.487	0.491	0.485	0.475	**0.495**	0.486	0.473	0.463	0.482
EW	**0.329**	0.291	0.307	0.238	0.321	0.304	0.297	0.291	0.297
SOL	0.081	0.157	**0.235**	0.134	0.118	0.132	0.139	0.115	0.139
STC	0.272	0.283	**0.318**	0.304	0.283	0.288	0.299	0.316	0.295
TP	**0.268**	0.253	0.220	0.253	0.235	0.231	0.218	0.233	0.239
TPF	**0.314**	0.306	0.247	0.295	0.280	0.280	0.292	0.273	0.286
Wisconsin									0.579
CR	**0.508**	0.429	0.494	0.401	0.436	0.409	0.415	0.389	0.435
DTP	**0.792**	0.764	0.778	0.744	0.766	0.754	0.747	0.722	0.758
DTS	**0.826**	0.810	0.788	0.766	0.812	0.804	0.770	0.753	0.791
EL	0.799	0.800	0.800	0.807	0.816	0.813	**0.821**	0.817	0.809
ES	0.505	0.558	0.496	**0.506**	**0.560**	0.553	0.511	0.509	0.525
EW	0.654	0.588	**0.667**	0.568	0.568	0.528	0.589	0.529	0.586
FLA	**0.462**	0.372	0.437	0.335	0.337	0.309	0.349	0.316	0.365
HAP	**0.692**	0.647	0.685	0.645	0.649	0.634	0.662	0.626	0.655
HP	**0.619**	0.609	0.609	0.593	0.603	0.598	0.594	0.582	0.601
KRN	**0.837**	0.830	0.818	0.811	0.832	0.826	0.814	0.805	0.822
RAP	0.390	**0.450**	0.327	0.404	0.433	0.431	0.406	0.411	0.407
RT	0.254	0.187	**0.288**	0.210	0.170	0.133	0.202	0.190	0.204
TPF	0.718	0.726	0.727	0.725	**0.741**	0.733	0.735	0.725	0.729
TXT	0.381	**0.443**	0.407	0.406	**0.443**	0.441	0.397	0.417	0.417

CR, color rate; CUR, curvature; DTP, days to pollination; DTS, days to silking; EL, ear length; EH, ear height; ES, ear shape; EW, ear width; FLA, flavor; HAP, husk appearance; HP, husk protection; KRN, kernel row number; PH, plant height; RAP, Row appearance; RT, rating; SOL, solidity; STC, stand count; TP, taper; TPF, tip fill; TXT, texture; AG, GBLUP single-trait with additive effect; ADG, GBLUP single-trait with additive effect and dominance effect; MAG, GBLUP multi-trait with additive effect; MADG, GBLUP multi-trait with additive effect and dominance effect; AR, RKHS single-trait with additive effect; ADR, RKHS single-trait with additive effect and dominance effect; MAR, RKHS multi-trait with additive effect; MADR, RKHS multi-trait with additive effect and dominance effect.

CV0 = Prediction of tested hybrids in untested environments. Bolded numbers represent the model with the highest performance for each trait.

### 3.4 Within-site across-year: new environments and new hybrid predictions (CV00)

**Figure 4:**
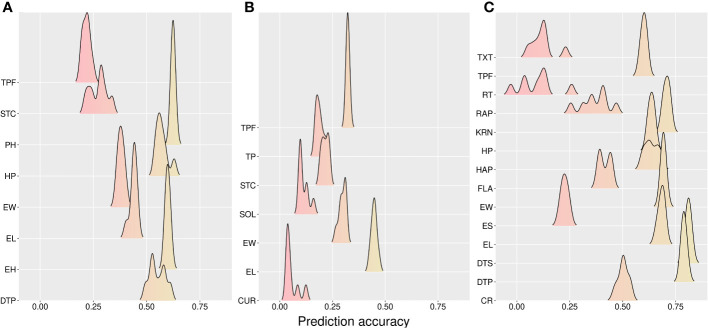
Accuracy for prediction across models of new hybrids in new environments (CV00). **(A)** California, **(B)** Florida, and **(C)** Wisconsin sites. CR, color rate; CUR, curvature; DTP, days to pollination; DTS, days to silking; EL, ear length; EH, ear height; ES, ear shape; EW, ear width; FLA, flavor; HAP, husk appearance; HP, husk protection; KRN, kernel row number; PH, plant height; RAP, row appearance; RT, rating; SOL, solidity; STC, stand count; TP, taper; TPF, tip fill; TXT, texture.

The results from CV00 were consistent with the previous insights for CV0. However, with a slightly lower prediction accuracy compared with CV0 (CA: 0.457, FL: 0.234, and WI: 0.520) (**Table 3**). Furthermore, multi-trait models were better in CA (3.19%), FL (2.46%), and WI (0.70), whereas the A model overperformed the AD model in CA (1.73%), FL (3.66%) and WI (2.52%). The best mean predictability across models was for the DTP trait in the WI site (0.82), whereas the lower value was found for CUR (0.124) (**Figure 4**).

**Table 3:** Prediction accuracy of within-site across-year hybrid prediction for California, Florida, and Wisconsin.

Trait	Model								Mean
	AG	ADG	MAG	MADG	AR	ADR	MAR	MADR	
California									0.457
DTP	0.583	**0.611**	0.526	0.495	0.578	0.557	0.521	0.534	0.551
EH	0.586	0.591	0.602	0.601	0.594	0.595	0.611	**0.612**	0.599
EL	0.437	0.446	0.404	0.434	**0.455**	0.454	0.437	0.442	0.439
EW	**0.403**	0.370	0.392	0.358	0.388	0.378	0.380	0.367	0.380
HP	0.559	0.578	0.590	**0.629**	0.539	0.547	0.558	0.568	0.571
PH	0.626	0.616	**0.633**	0.621	0.623	0.623	0.630	0.622	0.624
STC	**0.338**	0.309	0.288	0.212	0.288	0.278	0.247	0.229	0.274
TPF	**0.247**	0.199	0.223	0.194	0.231	0.217	0.223	0.202	0.217
Florida									0.234
CUR	0.036	0.051	**0.124**	0.085	0.034	0.034	0.044	0.038	0.056
EL	0.455	0.436	**0.470**	0.427	0.451	0.444	0.453	0.442	0.447
EW	**0.314**	0.294	0.311	0.266	0.311	0.309	0.289	0.287	0.298
SOL	0.092	0.127	**0.160**	0.128	0.100	0.099	0.096	0.098	0.113
STC	0.208	0.192	**0.235**	0.196	0.220	0.207	0.227	0.233	0.215
TP	**0.221**	0.190	0.168	0.205	0.191	0.175	0.181	0.172	0.188
TPF	0.318	0.324	0.319	0.327	0.321	0.319	0.322	**0.330**	0.323
Wisconsin									0.520
CR	0.505	0.503	0.461	0.478	0.533	**0.534**	0.501	0.503	0.502
DTP	0.789	0.794	0.797	**0.806**	0.783	0.781	0.787	0.778	0.789
DTS	0.821	**0.826**	0.807	0.816	0.811	0.808	0.800	0.791	0.810
EL	0.695	0.681	0.687	0.658	**0.698**	0.697	0.681	0.668	0.683
ES	0.215	0.216	0.242	0.231	0.218	0.199	**0.250**	0.234	0.226
EW	**0.706**	0.690	0.695	0.680	0.696	0.695	0.691	0.687	0.693
FLA	**0.447**	0.442	0.444	0.397	0.396	0.384	0.426	0.388	0.416
HAP	0.668	0.590	**0.685**	0.619	0.627	0.602	0.658	0.639	0.636
HP	0.626	0.640	**0.653**	0.646	0.620	0.619	0.634	0.647	0.636
KRN	0.712	0.720	0.695	0.689	0.719	**0.724**	0.694	0.706	0.707
RAP	0.397	**0.469**	0.255	0.360	0.412	0.410	0.313	0.348	0.371
RT	0.137	0.042	**0.259**	0.116	0.031	-0.029	0.129	0.095	0.098
TPF	0.610	0.597	**0.615**	0.605	0.595	0.582	0.609	0.587	0.600
TXT	0.134	0.097	**0.230**	0.129	0.076	0.051	0.130	0.114	0.120

CR, color rate; CUR, curvature; DTP, days to pollination; DTS, days to silking; EL, ear length; EH, ear height; ES, ear shape; EW, ear width; FL, flavor; HAP, husk appearance; HP, husk protection; KRN, kernel row number; PH, plant height; RAP, Row appearance; RT, rating; SOL, solidity; STC, stand count; TP, taper; TPF, tip fill; TXT, texture; Bolded numbers represent the model with the highest performance for each trait; AG, GBLUP single-trait with additive effect; ADG, GBLUP single-trait with additive effect and dominance effect; MAG, GBLUP multi-trait with additive effect; MADG, GBLUP multi-trait with additive effect and dominance effect; AR, RKHS single-trait with additive effect; ADR, RKHS single-trait with additive effect and dominance effect; MAR, RKHS multi-trait with additive effect; MADR, RKHS multi-trait with additive effect and dominance effect.

CV00 is the prediction of new hybrids in new environments.

### 3.5 Hybrid prediction accuracy for new environments (CV0) with information from all sites

In this cross-validation scheme, we used trait information for EL, EW, and TPF across all locations in 2020 to train the model. We aimed to predict traits in the 2021 locations. Models ADR (0.828), AG (0.648), and MAG (0.786) were best at predicting the traits EL, EW, and TPF, respectively (**Table 4** and **Supplementary material – Supplementary Table** **S7**). In addition, higher accuracies were found for EL as compared to EW and TPR. The mean prediction accuracy for all traits for FL21 presented a lower average value of 0.409, compared to 0.492 (CA21) and 0.72 (WI21). It is worth mentioning that this last site (WI21) excels in prediction accuracy for TPF traits compared with the other two environments (0.75 against 0.42 and 0.16 of CA21 and FL21).

**Table 4:** Prediction accuracy for across-site hybrid prediction.

Sites	AG	ADG	MAG	MADG	AR	ADR	MAR	MADR
EL								
**CA21**	0.657	0.675	0.675	0.701	0.676	0.678	**0.707**	0.699
**FL21**	0.417	0.433	0.421	**0.428**	0.407	0.412	0.418	0.425
**WI21**	0.783	0.823	0.790	0.807	0.819	**0.828**	0.821	0.813
EW								
**CA21**	0.371	**0.375**	0.365	0.364	0.367	0.365	0.355	0.363
**FL21**	0.392	0.396	0.393	0.382	0.402	**0.404**	0.398	0.396
**WI21**	**0.648**	0.605	0.644	0.617	0.621	0.603	0.625	0.601
TPF								
**CA21**	0.396	0.411	0.450	0.433	0.404	0.413	0.453	**0.465**
**FL21**	0.154	0.148	0.167	**0.179**	0.151	0.159	0.169	0.180
**WI21**	0.765	0.777	**0.786**	0.734	0.755	0.743	0.761	0.680

CA21, California site; year of 2021; FL21, Florida site; year of 2021; WI21, Wisconsin site; year of 2021; EL, ear length; EW, ear width; TPF, tip fill; AG, GBLUP single-trait with additive effect; ADG, GBLUP single-trait with additive effect and dominance effect; MAG, GBLUP multi-trait with additive effect; MADG, GBLUP multi-trait with additive effect and dominance effect; AR, RKHS single-trait with additive effect; ADR, RKHS single-trait with additive effect and dominance effect; MAR, RKHS multi-trait with additive effect. MADR, RKHS multi-trait with additive effect and dominance effect.

All the information from 2020 (CA20, FL20, and WI20) was used to train the model. The cross-validation scheme was the CV0 (tested hybrids in untested environments). Bolded numbers represent the model with the highest performance for each trait. The complete list with all site combinations is presented in the **Supplementary material – Supplementary Table S7**.

### 3.6 New hybrids for new environments (CV00) with information from all sites

The prediction of new hybrid performance in new environments varied for each trait and model used for the across-site CV00 scheme (**Table 5** and **Supplementary material – Supplementary Table S8**). The prediction accuracies for WI21 were better for all three traits when compared to FL21 and CA21. When we considered the prediction of FL21, the accuracies were lower, with the model MADR performing better for EL. When evaluating EW and TPF, we found the model ADG performed better. On the other hand, the best predictions came from WI21, where the AG model achieved higher accuracy for all three traits (0.641, 0.698, and 0.614, for EL, EW, and TPF traits).

**Table 5:** Prediction accuracy of across-site hybrid prediction.

Sites	AG	ADG	MAG	MADG	AR	ADR	MAR	MADR
EL								
**CA21**	**0.487**	0.466	0.470	0.486	0.496	0.484	0.497	0.493
**FL21**	0.398	0.414	0.381	0.418	0.405	0.413	0.402	**0.417**
**WI21**	**0.641**	0.620	0.611	0.605	0.638	0.615	0.619	0.579
EW								
**CA21**	0.411	**0.412**	0.399	0.408	0.408	0.409	0.402	**0.412**
**FL21**	0.368	**0.397**	0.360	0.365	0.379	0.370	0.376	0.372
**WI21**	**0.698**	0.688	0.697	0.694	0.690	0.691	0.695	0.686
TPF								
**CA21**	0.360	0.325	**0.415**	0.364	0.324	0.329	0.383	0.389
**FL21**	0.224	**0.414**	0.241	0.242	0.236	0.207	0.246	0.275
**WI21**	**0.614**	0.597	0.497	0.323	0.581	0.540	0.438	0.384

CA21, California site; year of 2021; FL21, Florida site; year of 2021; WI21, Wisconsin site; year of 2021; EL, ear length; EW, ear width; TPF, tip fill; AG, GBLUP single-trait with additive effect; ADG, GBLUP single-trait with additive effect and dominance effect; MAG, GBLUP multi-trait with additive effect; MADG, GBLUP multi-trait with additive effect and dominance effect; AR, RKHS single-trait with additive effect; ADR, RKHS single-trait with additive effect and dominance effect; MAR, RKHS multi-trait with additive effect; MADR, RKHS multi-trait with additive effect and dominance effect. MADR, RKHS multi-trait with additive effect and dominance effect.

Only the genotypes that were not assessed at the testing site were included in the training set from 2020 sites (CA20, FL20, and WI20). The cross-validation scheme was the CV00 (untested hybrids in untested environments). Bolded numbers represent the model with the highest performance for each trait. The complete list with all site combinations is presented in the **Supplementary material – Supplementary Table S7**.

## 4 Discussion

As the field of quantitative genomics evolves, tools for genomic selection have gained popularity. Genomic selection allows breeders to test new genotypes and predict their performance in untested environments. Genomic selection is proposed to boost different stages of a breeding program (Allier et al., 2019; Oliveira et al., 2020; Powell et al., 2020). For hybrid crops, the prediction of a potential cross from genotyped parents represents an important tool that ensures the best candidates will reach advanced field stages (Kadam et al., 2021; Oliveira et al., 2022). Routinely, sweet corn breeders assess hybrid performance by examining a set of target traits in several environments. Model selection and implementation of cross-validation schemes to optimize advanced field stages is still a challenge for the implementation of genomic selection for *in silico* hybrid prediction.

### 4.1 Model optimization for hybrid prediction

Historically, sweet corn breeders select for several breeding targets at once requiring complex decisions in the breeding program. For instance, the traits studied are related in complex ways, with correlations varying from highly positive to very negative values. In addition, trait heritabilities can vary greatly. This level of complexity directly affects model accuracy (Montesinos-López et al., 2016). It is common knowledge that in multi-trait models, traits with lower heritabilities can borrow information from traits with high heritabilities, once correlations are established, leading to an improvement in accuracy (Jia and Jannink, 2012; Mrode, 2014). However, our results indicated that little benefit is gained from multi-trait over single-trait models. In some cases, the use of a single-trait model can result in better performance overall, supporting findings from other crop species (Covarrubias-Pazaran et al., 2018; Oliveira et al., 2020; Sandhu et al., 2022). Two reasons are hypothesized for this result. First, the small sample size of our data could represent a weakness for the multi-trait model, since sample size can impact the multi-trait model performance (Schönbrodt and Perugini, 2013). The second factor is that lower or absence of significative correlations between traits with large differences in heritability, when combined in the same model, do not improve the prediction of traits with low heritability. An antagonistic example of this pattern can be found in the flavor and texture traits, with increasing for multi-trait models (CV1). Both traits have low heritability, and the prediction accuracy was also lower compared to other traits. However, the prediction accuracy values improved in models with multi-trait structure. This further illustrates that lower heritability traits can be the result of a trait that is difficult to measure, making it clearer that multi-trait models can improve the prediction of hard-to-measure traits.

Our results indicate that the addition of non-additive effects in the model did not improve the average prediction accuracy. However, including dominance in the models for tip fill, a target trait for sweet corn seems to have a positive impact. For hybrid prediction in field corn, it is known that non-additive effects play an important role. But the impact varies from trait to trait (Alves et al., 2019; Ferrão et al., 2020; Rogers et al., 2021). For instance, Coelho et al. (2020) estimate, in field corn, a coefficient of determination for the dominance effects of 0.32 (grain yield), 0.02 (ear length), 0.13 (ear height), and 0.05 (plant height). Whereas Nardino et al. (2020) demonstrate that the proportion between the general combining ability (GCA) and specific combing ability (SCA) varies from 0.92 to 7.26 among sites for yield-related traits. Together, the complexity and importance of dominance may vary between traits and will ultimately impact the implementation of a model with non-additive effects in hybrid prediction. On the other hand, the role dominance plays in sweet corn genomic selection is still emerging. Early evidence suggests that non-additive effects represent an important factor in the model, but these effects can also vary from one trait to another (Zystro et al., 2021a, b). For instance, Neto et al. (2022) indicate a significant impact of dominance on the SCA estimates in the performance of an F1 population, with a GCA/SCA ratio smaller than one unit for plant height, ear height, ear length, and kernel row number.

In Zystro et al. (2021a) the application of five-fold cross-validation (equivalent to our CV1 scheme) returns an average increase of 50% in trait prediction for the GBLUP model when comparing the A and AD models. However, on average, the results of our CV1 scheme with the inclusion of dominance slightly reduced the prediction accuracy.

The results demonstrated that conventional GBLUP models can be more advantageous over RKHS for hybrid performance in all schemes presented here (CV1, CV0, and CV00). The GBLUP kernels here explored in the model (A and D, for additive and dominance information) were constructed based on the realized relationship matrix, as proposed by VanRaden (2008), for the additive kernel, and on the proposal made by Vitezica et al. (2013) for the dominance kernel. Nonetheless, the enhancement of predictive performance is achievable through the construction of non-parametric matrices. It is advantageous to carefully select a kernel matrix that effectively encapsulates the inherent characteristics of the data, thereby leveraging and optimizing prediction accuracy.

In the case of RKHS, the kernel implicitly included non-additive effects (Gianola and Van Kaam, 2008) such as epistasis (E). Then, the markers matrix coding (for A and D kernels) makes it possible to capture epistatic effects, such as additive-by-additive and dominance-by-dominance epistatic effects. Therefore, the performance of RKHS and GBLUP models has been shown to vary among crops (Kadam and Lorenz, 2019; Lopez-Cruz et al., 2021), and similar performance and/or minimal advantage is reported by AD-RKHS models over AD- or ADE-GBLUP (Lyra et al., 2018; Alves et al., 2021). For sweet corn hybrid prediction, the conventional GBLUP seems to represent the optimum model and is robust enough for several traits with a complex trait architecture, even under the simplest assumptions.

### 4.2 Implementation of cross-validation schemes in sweet corn hybrid prediction

We compared the implementation of different cross-validation (CV) schemes as outlined in Burgueño et al. (2012) and Jarquin et al. (2018). It is worth mentioning that genomic selection can contribute to a breeding program by predicting unobserved genotypes and/or environments. By that, the application of this tool may reduce the number of field trials and ensure potential candidates (hybrids in our study) advance through the trialing stages. The CV1 scheme hypothesizes that the information of untested hybrids in a tested environment is used to predict non-yet-observed hybrids. The results of CV1 had higher prediction accuracy compared with the other schemes, once all the information from genotypes was made available, but the prediction set, in the target environment. The prediction performance of CV1 exploits the genetic correlation between the training set and the prediction set (Jarquin et al., 2018), by using the information of genetic materials observed in the same environment to predict unobserved materials. As the set of hybrids here studied came from 62 individual lines (hybrid parents), the relatedness among individuals that compose the CV partition (training and prediction set of individuals) boosted the prediction accuracy of the CV1 scheme. In addition, the FL site had the lowest average performance for CV1, being outperformed by CA and WI sites. In this case, the number of hybrids assessed in 2020 in FL was significantly higher than those in CA and WI (418, 246, and 236, respectively), which should have built a stronger correlation between training and prediction sets, leading to higher accuracy. However, we hypothesize that, as the crosses include material with a temperate background (Wisconsin lines), the high pressure of the FL site (tropical climate, with high impact of diseases and pests), may have affected the hybrid performance, and ultimately, the model prediction accuracy for FL site. This pattern of germplasm by environment interaction can be seen in the other CV schemes.

In a sweet corn breeding program, selected hybrid combinations are sequentially tested across different years and environments, before release (Peixoto et al., 2023). First, new hybrids are generated from the crossing of many advanced lines against a few testers and assessed in a few environments. The parents/lines of the best-performing hybrids are selected based on the general and specific combining abilities. These parents/lines then proceed to the next stage where they are crossed to a larger number of testers and assessed across more environments. Again, selection is made based on both (general and specific combining abilities) to identify the best two parents/lines. These two parents/lines are crossed by even more testers and planted in large environment trials to evaluate their performance.

The CV0 scheme can predict how genetic material will perform in an unknown environment or a targeted number of unknown environments based on known performance in a tested location. For a sweet corn breeding program, this can help predict how a hybrid will perform in the second and third field hybrid stages. The CV00 scheme prediction can aid the *in silico* prediction of the best hybrid combinations for the first stage of testcrossing. Ultimately, it can guarantee that the best hybrid combinations are included in the first advanced trials. However, CV00 can be the hardest hybrid set to predict with its lack of information for both new hybrids and environments (Burgueño et al., 2012; Jarquin et al., 2018). Furthermore, it had the lowest accuracy compared with other schemes, as reported in other studies (Jarquin et al., 2018; Vieira et al., 2022). Then, CV00 prediction for sweet corn hybrids reinforces those hypotheses, given that the average accuracy was lower than the other schemes. However, for several traits, the predictability achieved good levels of accuracy (*i*.*e*., PH in the CA site: 0.62, EL in the FL site: 0.44, DTS in the WI site: 0.81). These findings increase our understanding of sweet corn performance over a CV00 and increase our confidence in its implementation in the breeding program. Compared to CV0 and CV00, the CV0 returned higher prediction accuracy, this was expected given that the genetic material in the CV0 (hybrids) was already assessed in the site in the previous year.

The three stages of hybrid prediction deal with the disturbance factors caused by the presence of the G×E interaction. Dealing with the G×E interaction is a challenge in hybrid prediction (Schrag et al., 2019; Rogers et al., 2021). This was observed in our results as the prediction of across-sites was lower compared to the within-site across-year prediction, for the CV0 and CV00 schemes. In advanced field stages, the presence of the G×E interaction can cause differences in field evaluation and, ultimately in the ranking of genotypes. This will impact the composition of the selected set of parents. Considering only the across-site prediction, when the information of all sites was used in the training set, the accuracy of the prediction increased. The inclusion of this type of data represents a way to circumvent the G×E interaction in hybrid prediction in sweet corn.

### 4.3 Future directions

This was the first effort to increase the knowledge of hybrid performance through genomic prediction in a multi-trait framework and using different kernel structures in sweet corn. Some traits, such as flavor, color rating, and texture, are related to consumer preferences. Such traits demand more systematic approaches for enhancement based on genetic performance aligned with consumer preferences. For these traits, we should use available tools to guide breeding targets. Future directions to enhance consumer-related traits can be done with the application of algorithms to predict consumer preferences based on genomics and metabolite data (Colantonio et al., 2022), which can bring a new understanding of consumer inclinations, such as flavor perception. Alternatively, the use of near-infrared technology can add to the inference of the relationship between lines. This adds to the relationship matrices that can capture patterns that SNP-based matrices cannot, allowing for phenomic selection (Krause et al., 2019). Another strategy that adds up for a breeding program that accounts for several traits at a time is the prediction with sparse phenotyping (Bhatta et al., 2020; Isidro y Sánchez and Akdemir, 2021). For instance, those authors found that we can improve the prediction ability of predictions of non-measured traits using the genomic model for borrowing information among traits. Sweet corn, which would be an alternative, especially for consumer-related traits, seemed as hardest to measure.

Furthermore, aiming to optimize the testcross phases, sparse testing designs should be considered (Jarquin et al., 2020; Crespo-Herrera et al., 2021; Persa et al., 2023). These authors showed that this methodology has the potential to substantially save resources by optimizing the genotypes and environments explored in trials, by accounting for the G×E interaction. Thus, we can enhance a sweet corn breeding program by considering a sparse design in combination with hybrid prediction models. Combining these techniques can also make the program more feasible.

Several tools can be used to increase model prediction. We can leverage the quality of the phenotypic data collected in the testcross stages with high-throughput phenotyping tools, which have already been demonstrated to boost genetic gains in sweet corn breeding programs (Peixoto et al., 2023). Also, a program can take advantage of environmental covariables to build a covariance relationship matrix among trials (Bandeira e Sousa et al., 2017; Gevartosky et al., 2023). However, these environmental kernels seem to be dependent on germplasm, environment, and traits (Costa-Neto et al., 2021; Rogers and Holland, 2022). Another good alternative is the application of crop growth models (Cooper and Messina, 2021), which deal with the G×E interaction, largely identified in the testing phase of the sweet corn program.

## 5 Conclusion

For the first time, our study presents a comprehensive and detailed application of genomic selection exploring several traits of interest as a tool for hybrid prediction in a sweet corn breeding program. In addition, we investigated several statistical and genetic aspects, and we improved the knowledge of several traits in a sweet corn breeding program. Multi-trait models did not perform well in comparison with the single-trait counterparts, and, even though a small sample size could have impacted the models’ performance, it improved the prediction for only a few traits with small heritability. We also found that models including additive and dominance aspects presented an improved performance, however slightly, for many traits. The most prominent inference was for GBLUP models, which outperformed RKHS in all scenarios and is recommended as a standard model for sweet corn prediction. Hybrid prediction through genomic selection has the potential to improve sweet corn breeding and different prediction scheme outcomes can highlight the best strategy to be used in different stages of the breeding program.

## Data availability statement

The data presented in the study are deposited in the https://github.com/Resende-Lab/Peixoto_Sweet_corn_hybrid_prediction. repository.

## Author contributions

MP: Writing – original draft, Formal analysis, Methodology, Software. KL: Writing – review & editing, Data curation, Investigation. DJ: Writing – review & editing, Investigation, Software. PF: Writing – review & editing, Data curation. JZ: Writing – review & editing, Data curation. WT: Writing – review & editing, Data curation, Investigation. LB: Writing – review & editing, Investigation, Supervision. MR: Funding acquisition, Methodology, Project administration, Investigation, Resources, Writing – review & editing.
