# Supplementary material for: Utilizing genomic prediction to boost hybrid performance in a sweet corn breeding program
Source: Front Plant Sci. 2024 Apr 25;15:1293307. doi: 10.3389/fpls.2024.1293307 (PMC11080654; doi:10.3389/fpls.2024.1293307)
Supplement: Supplementary file 1 [file DataSheet_1.pdf]

Supplementary Material

1 Supplementary Figures and Tables

1.1 Supplementary Tables

Supplementary Table S1. Traits measured in each of the six environments here assessed. FL20: Florida site, 2020. FL21: Florida site, 2021. CA20: California site, 2020. CA21: California site, 2021. WI20: Wisconsin site, 2020. WI21: Wisconsin site, 2021.

	Environments					
Traits	FL20	FL21	CA20	CA21	WI20	WI21
EL	√	√	√	√	√	√
EW	√	√	√	√	√	√
TPF	√	√	√	√	√	√
STC	√	√	√	√	-	-
DTP	-	-	√	√	√	√
HP	-	-	√	√	√	√
KRN	√	-	-	-	√	√
PH	-	-	√	√	√	-
EH	-	-	√	√	√	-
SOL	√	√	-	-	-	-
TP	√	√	-	-	-	-
CUR	√	√	-	-	-	-
DTS	-	-	-	-	√	√
HAP	-	-	-	-	√	√
RAP	-	-	-	-	√	√
ES	-	-	-	-	√	√
CR	-	-	-	-	√	√
FLA	-	-	-	-	√	√
TXT	-	-	-	-	√	√
RT	-	-	-	-	√	√

√: indicates that the trait was measured at the respective site. EL: ear length. EW: ear width. TPF: tip fill. STC: stand count. DTP: days to pollination. HP: husk protection. KRN: kernel row number. PH: plant height. EH: ear height. SOL: solidity. TP: taper. CUR: curvature. DTS: days to silking. HAP: husk appearance. RAP: Row appearance. ES: ear shape. CR: color rate. FLA: flavor. TXT: texture. RT: rating.

Supplementary Table S2. Likelihood ratio test for the genotypic effect for the traits assessed in six environments for the individual analyses. FL20: Florida site, 2020. FL21: Florida site, 2021. CA20: California site, 2020. CA21: California site, 2021. WI20: Wisconsin site, 2020. WI21: Wisconsin site, 2021. * Significative and [na] non-significative by LRT test at 5% of probability and 1 degree of freedom.

LRT test for genotypic effect						
**Traits**	**FL20**	**FL21**	**CA20**	**CA21**	**WI20**	**WI21**
**CR**	-	-	-	-	0.2073*	0.4036*
**CUR**	9.4606*	27.5076 [na]	-	-	-	-
**DTP**	-	-	0.5445*	3.7875*	4.1053*	4.1928*
**DTS**	-	-	-	-	4.1662*	5.8363*
**EH**	-	-	145.6411*	21.7719*	104.9425*	-
**EL**	0.9345*	8.8201*	3.2375*	1.8987*	1.8788*	1.2911*
**ES**	-	-	-	-	0.3511*	0.2238*
**EW**	0.0344*	0.0576*	0.0718*	0.0672*	0.0412*	0.0495*
**FLA**	-	-	-	-	0.0188 [na]	0.0784*
**HAP**	-	-	-	-	0.3368*	0.2411*
**HP**	-	-	0.3246*	0.3350*	1.0397*	0.6585*
**KRN**	1.9737*	-	-	-	2.9495*	2.7128*
**PH**	-	-	320.2704*	96.2332*	223.4988*	-
**RAP**	-	-	-	-	0.1713*	0.0968*
**RT**	-	-	-	-	0.2637*	0.1230*
**SOL**	0.0001*	0.0009 [na]	-	-	-	-
**STC**	127.3940*	650.5065*	187.3044*	377.6829*	-	-
**TP**	35.1331*	99.6853*	-	-	-	-
**TPF**	0.0006*	0.0004*	1.3500*	0.7114*	1.0770*	0.9107*
**TXT**	-	-	-	-	0.14268*	0.14385*

EL: ear length. EW: ear width. TPF: tip fill. STC: stand count. DTP: days to pollination. HP: husk protection. KRN: kernel row number. PH: plant height. EH: ear height. SOL: solidity. TP: taper. CUR: curvature. DTS: days to silking. HAP: husk appearance. RAP: Row appearance. ES: ear shape. CR: color rate. FLA: flavor. TXT: texture. RT: rating.

Supplementary Table S3. Variance components for and Likelihood ratio test for the block effect for the traits assessed in three environments for the individual analyses. FL20: Florida site, 2020. FL21: Florida site, 2021. CA20: California site, 2020. * Significative and [na] non-significative by LRT test at 5% of probability and 1 degree of freedom.

LRT test for block effect			
**Trait**	**FL20**	**FL21**	**CA20**
**CUR**	0.0000 [na]	16.7827 [na]	-
**DTP**	-	-	0.0000 [na]
**EH**	-	-	29.3757*
**EL**	0.0154 [na]	0.0388 [na]	0.0000 [na]
**EW**	0.0009*	0.0000 [na]	0.0211*
**HP**	-	-	0.0063 [na]
**KRN**	0.0000 [na]	-	-
**PH**	-	-	63.3606*
**SOL**	0.0000 [na]	0.0001 [na]	-
**STC**	1.4495 [na]	11.8974 [na]	1.4317 [na]
**TP**	1.4627*	14.0605 [na]	-
**TPF**	0.0000*	0.0000 [na]	0.0257 [na]

CUR: curvature. DTP: days to pollination. EH: ear height. EL: ear length. HP: husk protection. KRN: kernel row number. PH: plant height. SOL: solidity. STC: stand count. TP: taper. TPF: tip fill.

Supplementary Table S4. Phenotypic mean and estimates of traits heritability and for the California site in 2020 and 2021 individual analyses.

California - 2020				
**Trait**	**Abbreviation**	**Phenotypic mean**	**Heritability**	**Standard error**
Stand count	STC	78.04 (4-100)	0.77	0.026
Days to pollination	DTP	33.63 (55-73)	0.45	0.117
Husk protection	HP	1.84 (1.0-3.66)	0.67	0.036
Ear width	EW	4.655 (3.0-6.0)	0.37	0.056
Ear length	EL	20.11 (14.0-26.0)	0.80	0.024
Tipfill	TPF	2.97(1.0-5.0)	0.68	0.036
Plant heigh	PH	154.7 (95.0-225.0)	0.65	0.054
Ear heigh	EH	70.62 (35.0-115.0)	0.63	0.053

California - 2021				
**Trait**	**Abbreviation**	**Phenotypic mean**	**Heritability**	**Standard error**
Stand count	STC	48.5 (0-100)	0.78	0.053
Days to pollination	DTP	65.98 (61-71)	0.61	0.082
Husk protection	HP	3.086 (1.0-5.0)	0.62	0.083
Ear width	EW	4.06 (3.0-5.0)	0.30	0.109
Ear length	EL	17.16 (12.6-22.0)	0.60	0.083
Tipfill	TPF	3.554 (1.0-5.0)	0.60	0.085
Plant heigh	PH	101.4 (60.0-115.0)	0.46	0.098
Ear heigh	EH	39.91 (15.0-75.0)	0.28	0.106

Supplementary Table S5. Phenotypic mean and estimates of traits heritability and for the Florida site in 2020 and 2021 individual analyses.

		**Florida - 2020**		
**Trait**	**Abbreviation**	**Phenotypic mean**	**Heritability**	**Standard error**
Stand count	STC	88.91 (16.66-100)	0.61	0.033
Kernel row number	KRN	15.42 (11.0-20.6)	0.65	0.030
Ear length	EL	12.56 (7.87-17-13)	0.52	0.038
Ear width	EW	3.60 (2.50-4.52)	0.54	0.036
Solidity	SOL	0.95 (0.84-0.97)	0.31	0.047
Taper	TP	77.07 (37.68-104.56)	0.41	0.043
Curvature	CUR	17.71 (5.35-62.69)	0.18	0.050
Tipfill	TPF	0.94 (0.76-0.99)	0.42	0.041

		**Florida - 2021**		
**Trait**	**Abbreviation**	**Phenotypic mean**	**Heritability**	**Standard error**
Stand count	STC	58.46 (4-100)	0.83	0.031
Tipfill	TPF	0.99 (0.97-0.99)	0.35	0.094
Ear length	EL	20.97 (14.31-32.00)	0.66	0.062
Ear width	EW	4.80 (3.74-7.34)	0.21	0.105
Solidity	SOL	0.82 (0.57-0.95)	0.16	0.097
Taper	TP	108.89 (63.23-160.86)	0.32	0.089
Curvature	CUR	50.40 (11.47-145.09)	0.04	0.097

Supplementary Table S6. Phenotypic mean and estimates of traits heritability for the Wisconsin site in 2020 and 2021 individual analyses.

		Wisconsin - 2020		
**Trait**	**Abbreviation**	**Phenotypic mean**	**Heritability**	**Standard error**
Days to pollination	DTP	53.27 (47-58)	0.82	0.021
Days to silking	DTS	55.55 (49-62)	0.81	0.022
Kernel row number	KRN	17.62 (11-24)	0.72	0.031
Husk appearence	HAP	3.839 (1-5)	0.30	0.060
Husk protection	HP	2.828 (0-5)	0.65	0.038
Ear width	EW	4.5 (2-5.4)	0.41	0.054
Ear length	EL	20.34 (15-26)	0.72	0.032
Tipfill	TPF	3.276 (1-5)	0.73	0.030
Row appearence	RAP	3.648 (1-5)	0.38	0.056
Ear shape	ES	3.539 (1-5)	0.49	0.050
Color rate	CR	3.309 (2-5)	0.39	0.060
Flavor	FLA	3.551 (1-5)	0.05	0.065
Texture	TXT	2.843 (1-5)	0.31	0.059
Rating	RT	2.807 (1-4)	0.47	0.051
Plant heigth	PH	192.5 (130-265)	0.67	0.036
Ear heigth	EH	77.46 (5-125)	0.43	0.053

		Wisconsin - 2021		
**Trait**	**Abbreviation**	**Phenotypic mean**	**Heritability**	**Standard error**
Days to pollination	DTP	55.17 (49-65)	0.74	0.060
Days to siling	DTS	57.04 (51-67)	0.72	0.064
Kernel row number	KRN	16.9 (10-22)	0.70	0.067
Husk appearence	HAP	3.81 (2-5)	0.24	0.106
Husk protection	HP	3.621 (1-5)	0.68	0.071
Ear weigth	EW	4611 (3.8-5.2)	0.52	0.092
Ear length	EL	19.62 (16-23)	0.75	0.058
Tipfill	TPF	3.791 (1-5)	0.69	0.070
Row appearence	RAP	3.748 (2-5)	0.32	0.105
Ear shape	ES	3.73 (1-5)	0.44	0.100
Color rate	CR	3.072 (1-5)	0.58	0.088
Flavor	FLA	3.522 (2-5)	0.21	0.106
Texture	TXT	2.896 (1-4)	0.35	0.105
Rating	RT	3.026 (1-5)	0.24	0.106

Supplementary Table S7. Prediction accuracy of across-sites hybrids prediction for the sites of California, Florida, and Wisconsin. EL: ear length. EW: ear width. TPF: tip fill. Here, the information from 2020 (CA20, FL20, and WI20) was used to training the model. The cross-validation scheme was the CV0 (tested hybrids in untested environments). FLWICA: training set combines CA20, FL20, and WI20 to predict CA21 site. FLCA: training set combines CA20 and FL20 to predict CA21 site. WICA: training set combines CA20 and WI20 to predict CA21 site. CAWIFL: training set combines CA20, FL20, and WI20 to predict FL21 site. CAFL: training set combines CA20 and FL20 and to predict FL21 site. WIFL: training set combines WI20 and CA20 to predict FL21 site. CAFLWI: training set combines CA20, FL20, and WI20 to predict WI21 site. CAWI: training set combines CA20 and WI20 and to predict WI21 site. FLWI: training set combines FL20 and WI20 to predict WI21 site.

TPE	AG	ADG	MAG	MADG	AR	ADR	MAR	MADR	Mean
				**EL**					0.598
**FLWICA**	0.657	0.675	0.675	0.701	0.676	0.678	**0.707**	0.699	0.683
**FLCA**	0.600	0.613	0.611	0.671	0.636	0.602	0.663	**0.668**	0.633
**WICA**	**0.467**	0.457	0.450	0.391	0.466	0.440	0.377	0.430	0.435
**CAWIFL**	0.417	**0.433**	0.421	0.428	0.407	0.412	0.418	0.425	0.420
**CAFL**	0.445	**0.450**	0.418	0.409	0.433	0.424	0.405	0.407	0.424
**WIFL**	**0.467**	0.457	0.450	0.391	0.466	0.440	0.377	0.430	0.435
**CAFLWI**	0.783	0.823	0.790	0.807	0.819	**0.828**	0.821	0.813	0.811
**CAWI**	0.840	0.853	0.833	0.838	0.859	**0.855**	0.848	0.850	0.847
**FLWI**	0.683	0.734	0.616	0.668	0.755	**0.759**	0.669	0.697	0.698
				**EW**					0.465
**FLWICA**	0.371	**0.375**	0.365	0.364	0.367	0.365	0.355	0.363	0.365
**FLCA**	**0.425**	0.416	0.404	0.392	0.406	0.400	0.390	0.377	0.401
**WICA**	**0.385**	0.379	0.383	0.371	0.383	0.383	0.379	0.374	0.380
**CAWIFL**	0.392	0.396	0.393	0.382	0.402	**0.404**	0.398	0.396	0.395
**CAFL**	0.387	0.379	0.379	0.369	0.396	**0.397**	0.394	0.382	0.385
**WIFL**	**0.385**	0.379	0.383	0.371	0.383	0.383	0.379	0.374	0.380
**CAFLWI**	**0.648**	0.605	0.644	0.617	0.621	0.603	0.625	0.601	0.621
**CAWI**	**0.636**	0.595	0.615	0.551	0.581	0.577	0.566	0.518	0.580
**FLWI**	0.693	0.652	**0.729**	0.696	0.659	0.630	0.697	0.671	0.678
				**TPF**					0.378
**FLWICA**	0.396	0.411	0.450	0.433	0.404	0.413	0.453	**0.465**	0.428
**FLCA**	0.288	0.262	**0.326**	0.218	0.189	0.174	0.240	0.220	0.240
**WICA**	0.090	0.095	0.141	0.146	0.119	0.138	**0.161**	0.159	0.131
**CAWIFL**	0.154	0.148	0.167	0.179	0.151	0.159	0.169	**0.180**	0.163
**CAFL**	0.193	0.196	0.246	0.267	0.196	0.214	0.254	**0.272**	0.230
**WIFL**	0.090	0.095	0.141	0.146	0.119	0.138	**0.161**	0.159	0.131

**CAFLWI**	0.765	0.777	**0.786**	0.734	0.755	0.743	0.761	0.680	0.750
**CAWI**	0.771	0.780	0.817	0.824	0.795	0.789	**0.826**	0.817	0.802
**FLWI**	0.722	**0.674**	0.529	0.333	0.623	0.531	0.445	0.377	0.529

AG: GBLUP single-trait with additive effect. ADG: GBLUP single-trait with additive effect and dominance effect. MAG: GBLUP multi-trait with additive effect. MADG: GBLUP multi-trait with additive effect and dominance effect. AR: RKHS single-trait with additive effect. ADR: RKHS single-trait with additive effect and dominance effect. MAR: RKHS multi-trait with additive effect. MADR: RKHS multi-trait with additive effect and dominance effect.

Supplementary Table S8. Prediction accuracy of across-site hybrids prediction for the sites of California, Florida, and Wisconsin. EL: ear length. EW: ear width. TPF: tip fill. Here, only the genotypes that were not assessed at the testing site were included in the training set from 2020 sites (CA20, FL20, and WI20). The cross-validation scheme was the CV00 (untested hybrids in untested environments). FLWICA: training set combines CA20, FL20, and WI20 to predict CA21 site. FLCA: training set combines CA20 and FL20 to predict CA21 site. WICA: training set combines CA20 and WI20 to predict CA21 site. CAWIFL: training set combines CA20, FL20, and WI20 to predict FL21 site. CAFL: training set combines CA20 and FL20 and to predict FL21 site. WIFL: training set combines WI20 and CA20 to predict FL21 site. CAFLWI: training set combines CA20, FL20, and WI20 to predict WI21 site. CAWI: training set combines CA20 and WI20 and to predict WI21 site. FLWI: training set combines FL20 and WI20 to predict WI21 site.

ENV	AG	ADG	MAG	MADG	AR	ADR	MAR	MADR	Mean
				EL					0.499
**FLWICA**	0.487	0.466	0.470	0.486	0.496	0.484	0.497	0.493	0.485
**FLCA**	0.482	0.450	0.360	0.492	0.488	0.462	0.481	0.457	0.459
**WICA**	0.463	0.470	0.455	0.477	0.486	0.484	0.473	0.465	0.471
**CAWIFL**	0.398	0.414	0.381	0.418	0.405	0.413	0.402	0.417	0.406
**CAFL**	0.426	0.415	0.432	0.404	0.422	0.439	0.454	0.414	0.426
**WIFL**	0.465	0.435	0.415	0.394	0.470	0.448	0.416	0.408	0.431
**CAFLWI**	0.641	0.620	0.611	0.605	0.638	0.615	0.619	0.579	0.616
**CAWI**	0.700	0.701	0.706	0.705	0.707	0.711	0.704	0.702	0.704
**FLWI**	0.527	0.482	0.487	0.456	0.523	0.509	0.483	0.470	0.492
				EW					0.482
**FLWICA**	0.411	0.412	0.399	0.408	0.408	0.409	0.402	0.412	0.408
**FLCA**	0.454	0.439	0.434	0.428	0.432	0.427	0.424	0.426	0.433
**WICA**	0.363	0.359	0.355	0.357	0.368	0.372	0.371	0.359	0.363
**CAWIFL**	0.368	0.397	0.360	0.365	0.379	0.370	0.376	0.372	0.373
**CAFL**	0.360	0.343	0.349	0.330	0.366	0.356	0.364	0.348	0.352
**WIFL**	0.354	0.350	0.350	0.350	0.365	0.358	0.354	0.352	0.354
**CAFLWI**	0.698	0.688	0.697	0.694	0.690	0.691	0.695	0.686	0.692
**CAWI**	0.680	0.670	0.652	0.643	0.673	0.671	0.658	0.624	0.659
**FLWI**	0.710	0.697	0.734	0.714	0.692	0.690	0.715	0.711	0.708
				TPF					0.383
**FLWICA**	0.360	0.325	0.415	0.364	0.324	0.329	0.383	0.389	0.361
**FLCA**	0.245	0.173	0.297	0.216	0.237	0.178	0.238	0.195	0.222
**WICA**	0.362	0.357	0.332	0.312	0.361	0.352	0.318	0.298	0.337
**CAWIFL**	0.224	0.414	0.241	0.242	0.236	0.207	0.246	0.275	0.261
**CAFL**	0.303	0.252	0.294	0.304	0.279	0.279	0.301	0.324	0.292
**WIFL**	0.085	0.087	0.139	0.183	0.102	0.107	0.169	0.156	0.128

**CAFLWI**	0.712	0.678	0.702	0.634	0.648	0.635	0.635	0.607	0.657
**CAWI**	0.709	0.693	0.706	0.691	0.685	0.676	0.699	0.685	0.693
**FLWI**	0.614	0.597	0.497	0.323	0.581	0.540	0.438	0.384	0.497

AG: GBLUP single-trait with additive effect. ADG: GBLUP single-trait with additive effect and dominance effect. MAG: GBLUP multi-trait with additive effect. MADG: GBLUP multi-trait with additive effect and dominance effect. AR: RKHS single-trait with additive effect. ADR: RKHS single-trait with additive effect and dominance effect. MAR: RKHS multi-trait with additive effect. MADR: RKHS multi-trait with additive effect and dominance effect.

1.2 Supplementary Figures

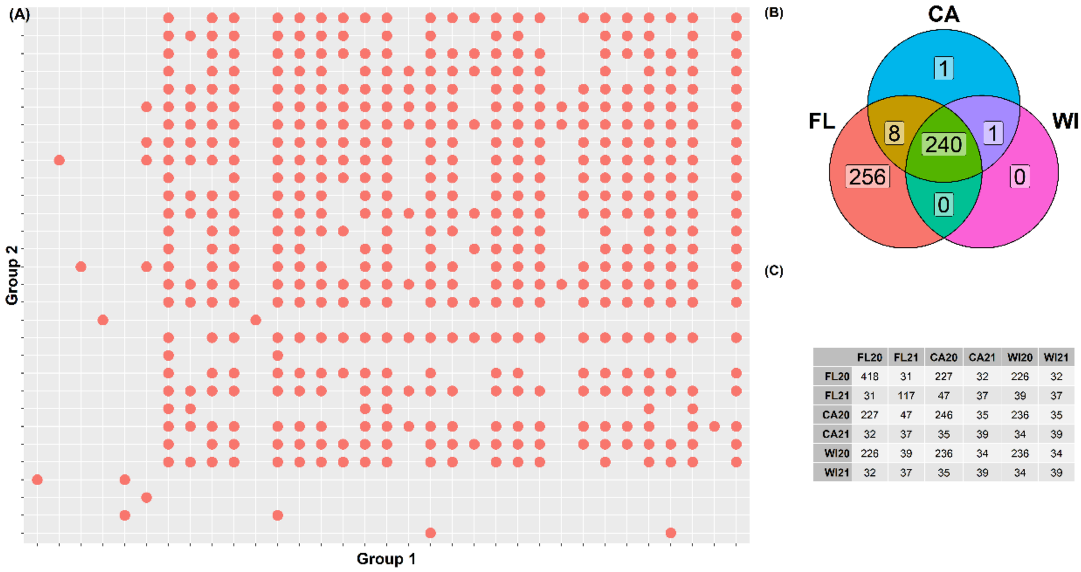

	FL20	FL21	CA20	CA21	WI20	WI21
FL20	418	31	227	32	226	32
FL21	31	117	47	37	39	37
CA20	227	47	246	35	236	35
CA21	32	37	35	39	34	39
WI20	226	39	236	34	236	34
WI21	32	37	35	39	34	39

Supplementary Figure S1. Summary of the hybrids and lines used in the study. (A) Schematic representation of the tested hybrid (506) parents by crossing the parentals (Group 1 and Group 2), where each red dot represents one cross between two parents. (B) A Venn diagram representing the number of hybrids planted that was shared among the three environments. FL: Florida site, CA: California site, WI: Wisconsin site. (C) Number of hybrids assessed at each environment (diagonal) and number of hybrids shared among the six environments (off-diagonal). FL20: Florida site, 2020. FL21: Florida site, 2021. CA20: California site, 2020. CA21: California site, 2021. WI20: Wisconsin site, 2020. WI21: Wisconsin site, 2021.

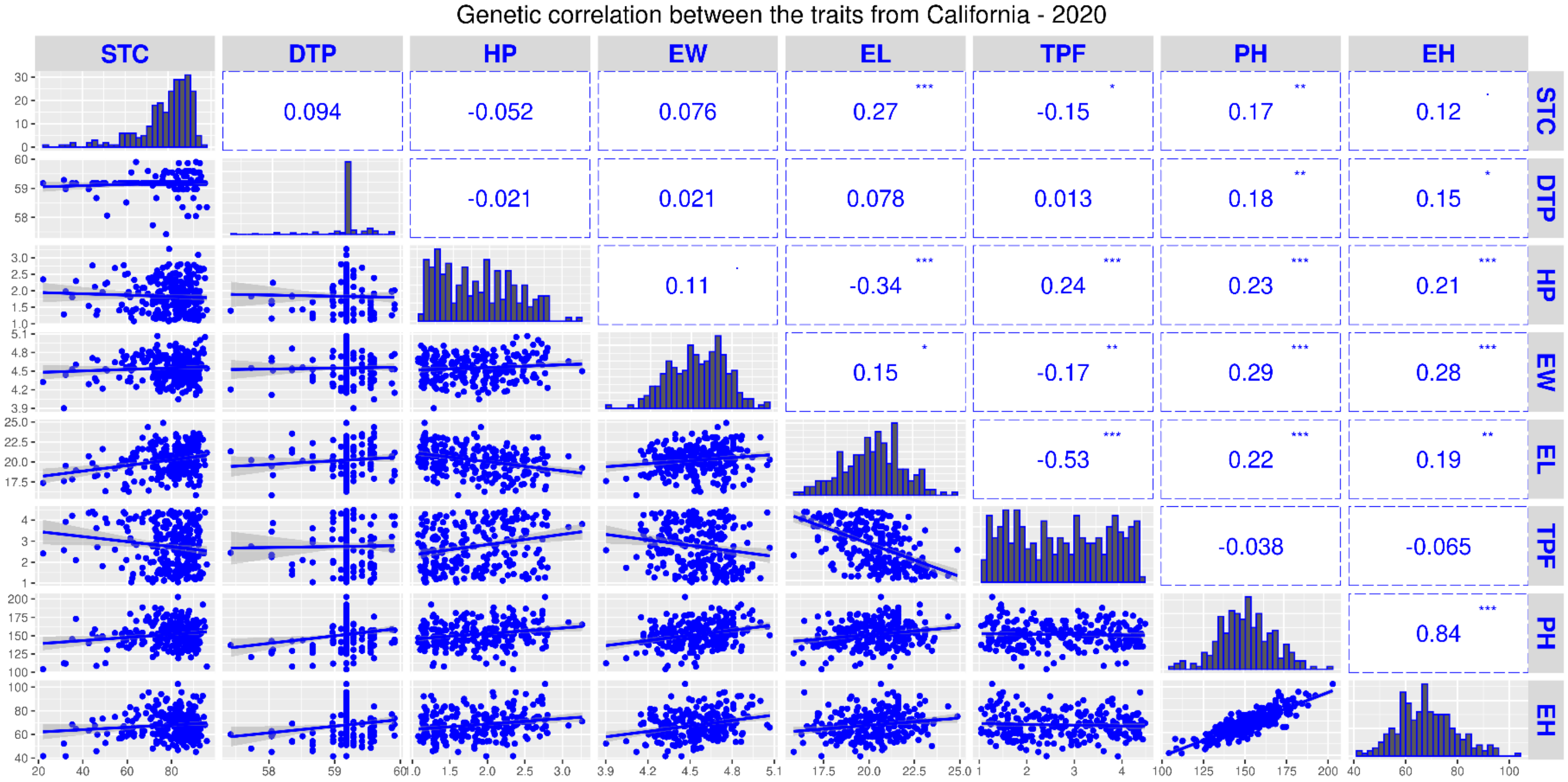

Supplementary Figure S2. Pearson correlations between vector of BLUPs for California 2020 environment. DTP: days to pollination. EH: ear heigth. EL: ear length. EW: ear width. HP: husk protection. PH: plant heigth. STC: stand count. TPF: tip fill. *,**,*** represents the significance of the correlations at 0.10, 0.05, and 0.01 levels.

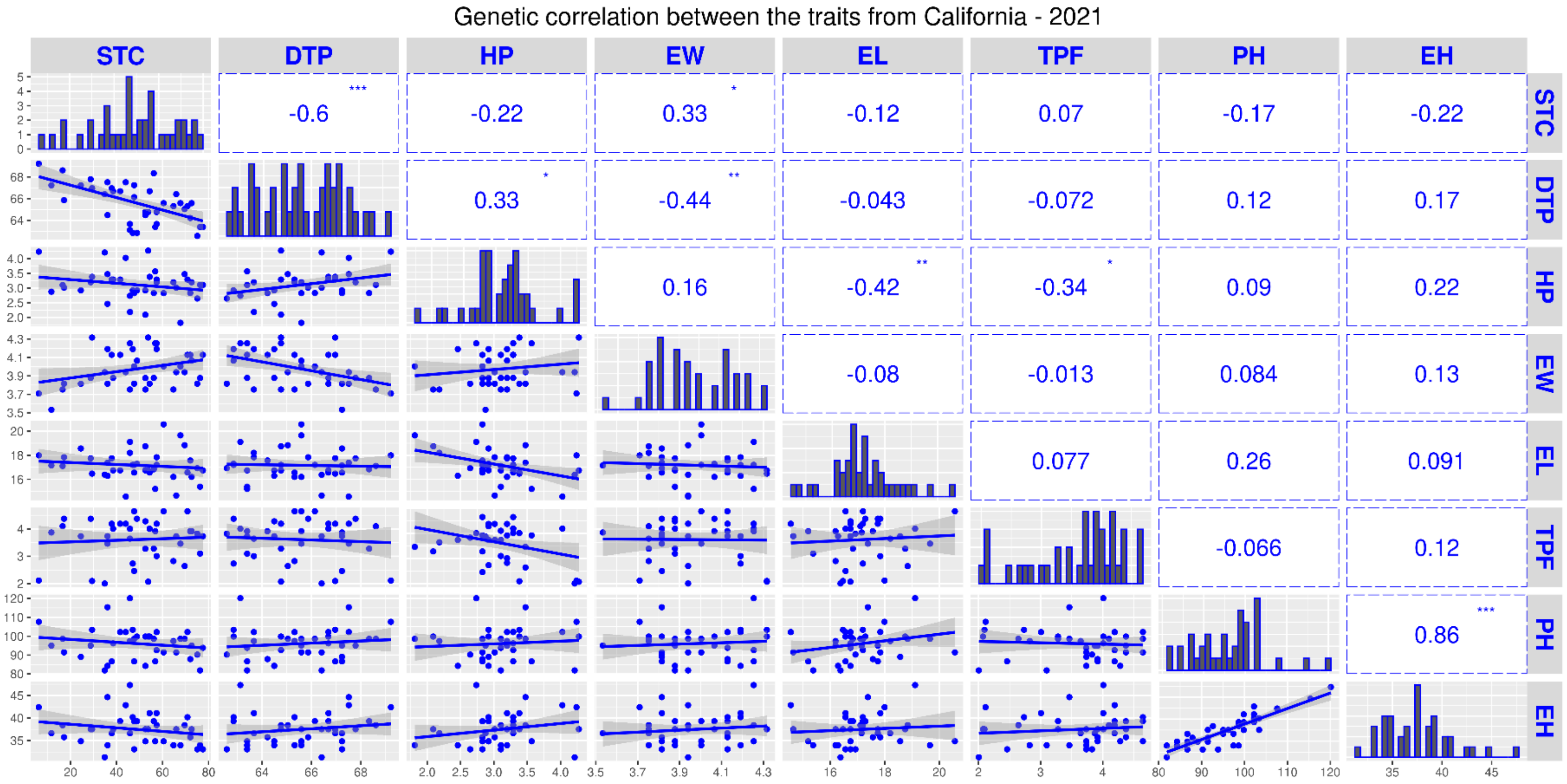

Supplementary Figure S3. Pearson correlations between vector of BLUPs for California 2021 environment. DTP: days to pollination. EH: ear heigth. EL: ear length. EW: ear width. HP: husk protection. PH: plant heigth. STC: stand count. TPF: tip fill. *,**,*** represents the significance of the correlations at 0.10, 0.05, and 0.01 levels.

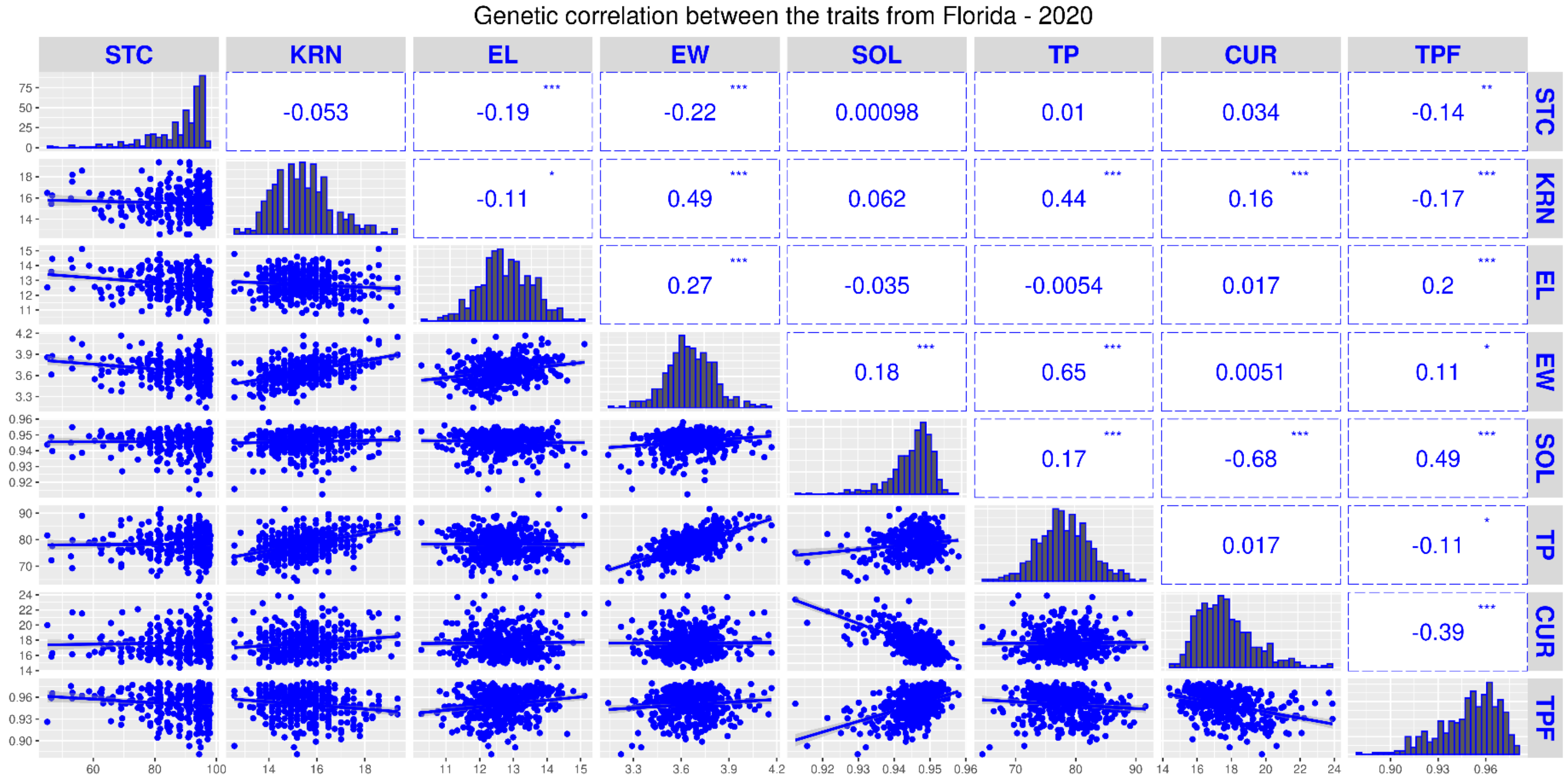

Supplementary Figure S4. Pearson correlations between vector of BLUPs for Florida 2020 environment. CUR: curvature. EL: ear length. EW: ear width. KRN: kernel row number. SOL: solidity, STC: stand count. TP: taper. TPF: tip fill. *,**,*** represents the significance of the correlations at 0.10, 0.05, and 0.01 levels.

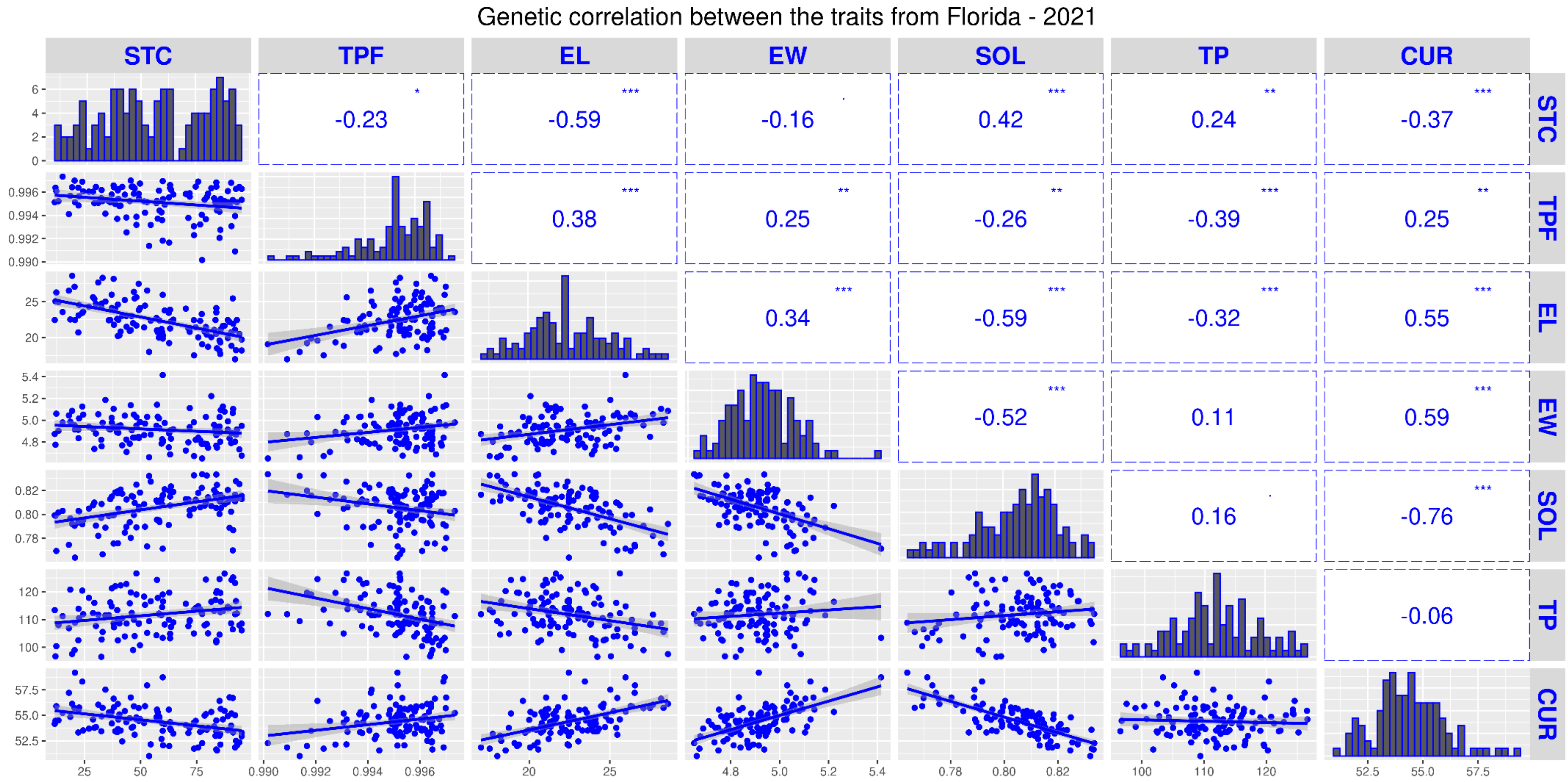

Supplementary Figure S5. Pearson correlations between vector of BLUPs for Florida 2021 environment. CUR: curvature. EL: ear length. EW: ear width. KRN: kernel row number. SOL: solidity, STD: stand count. TP: taper. TPF: tip fill. *,**,*** represents the significance of the correlations at 0.10, 0.05, and 0.01 levels.

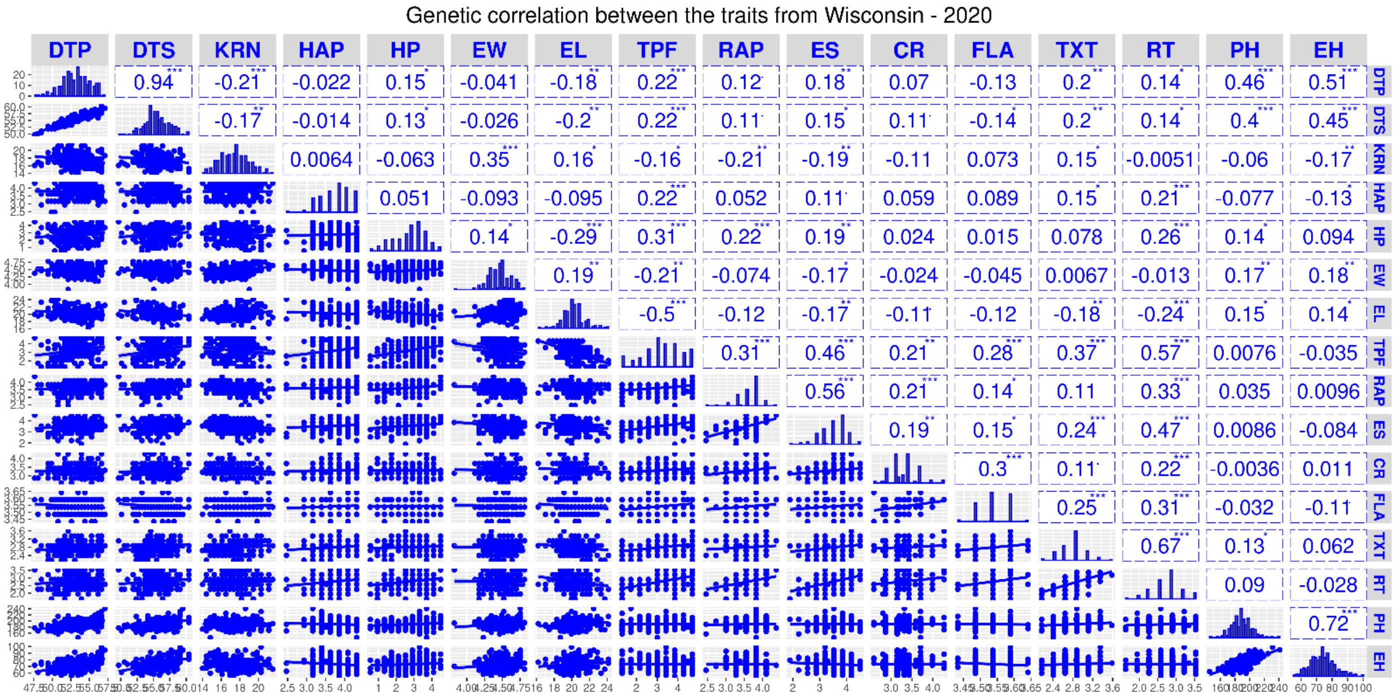

Supplementary Figure S6. Pearson correlations between vector of BLUPs for Wisconsin 2020 environment. CR: color rate. DTP: days to pollination. DTS: days to silking. EH: ear height. EL: ear length. ES: ear shape. EW: ear width. FLA: flavor. HAP: husk appearance. HP: husk protection. KRN: kernel row number. PH: plant height. RAP: Row appearance. RT: rating. TPF: tip fill. TXT: texture. *,**,*** represents the significance of the correlations at 0.10, 0.05, and 0.01 levels.

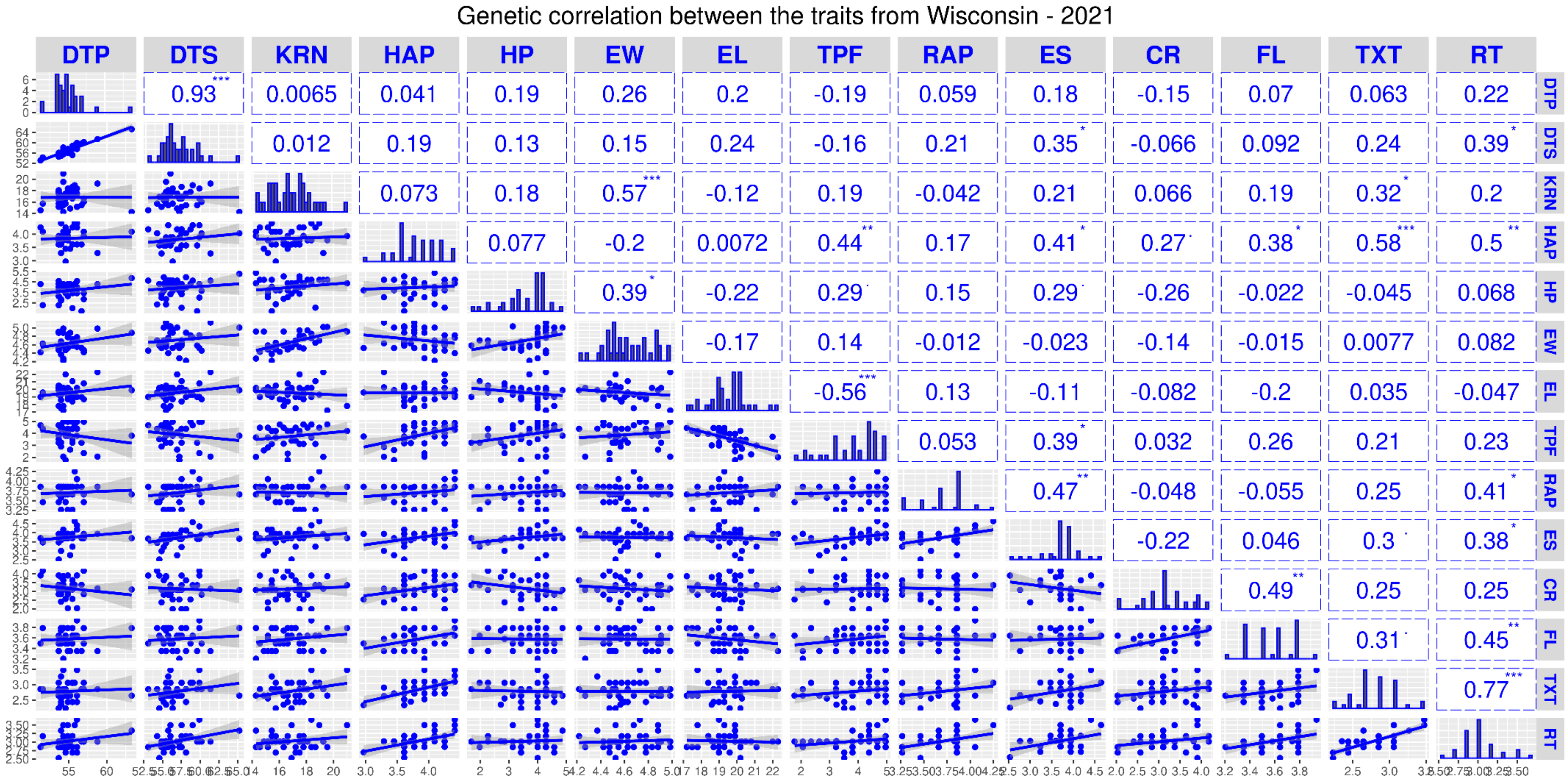

Supplementary Figure S7. Pearson correlations between vector of BLUPs for Wisconsin 2021 environment. CR: color rate. DTP: days to pollination. DTS: days to silking. EL: ear length. ES: ear shape. EW: ear width. FL: flavor. HAP: husk appearance. HP: husk protection. KRN: kernel row number. RAP: Row appearance. RT: rating. TPF: tip fill. TXT: texture. *,**,*** represents the significance of the correlations at 0.10, 0.05, and 0.01 levels.

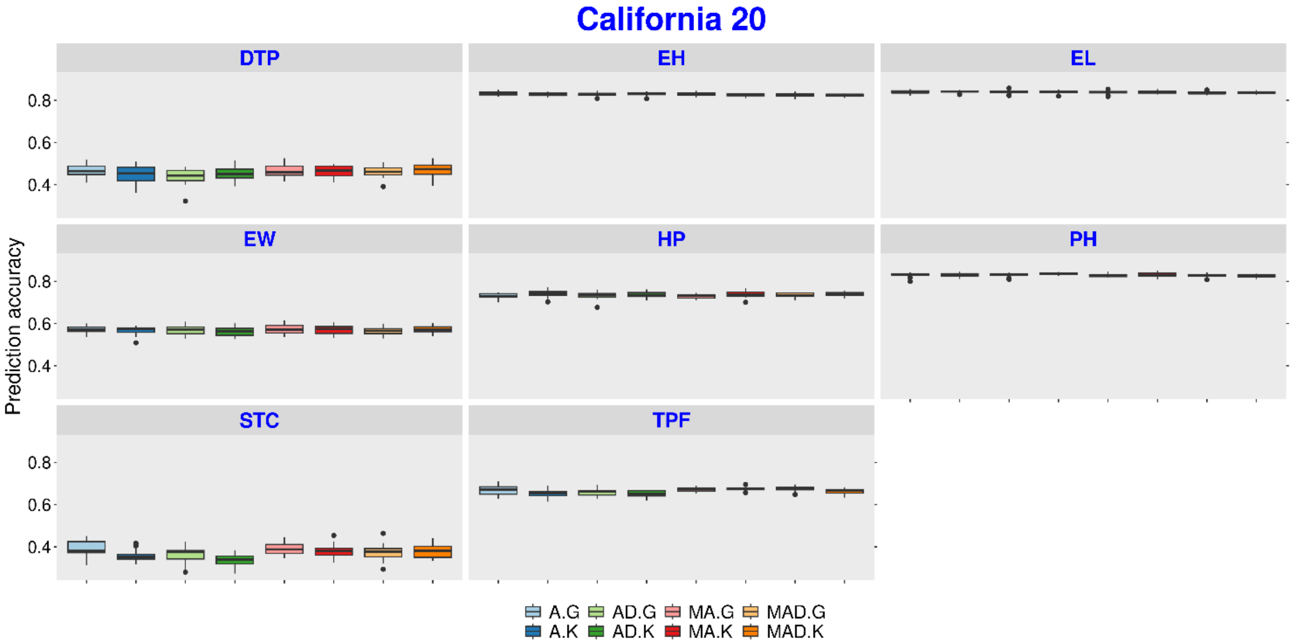

Supplementary Figure S8. Prediction accuracy for eigth traits using eigth four different models under GBLUP and RKHS predictions for California 2020 site via CV1 scheme. DTP: days to pollination. EH: ear heigth. EL: ear length. EW: ear width. HP: husk protection. PH: plant heigth. STC: stand count. TPF: tip fill. A.G: GBLUP single-trait with additive effect. AD.G: GBLUP single-trait with additive effect and dominance effect. MA.G: GBLUP multi-trait with additive effect. MAD.G: GBLUP multi-trait with additive effect and dominance effect. A.K: RKHS single-trait with additive effect. AD.K: RKHS single-trait with additive effect and dominance effect. MA.K: RKHS multi-trait with additive effect. MAD.K: RKHS multi-trait with additive effect and dominance effect.

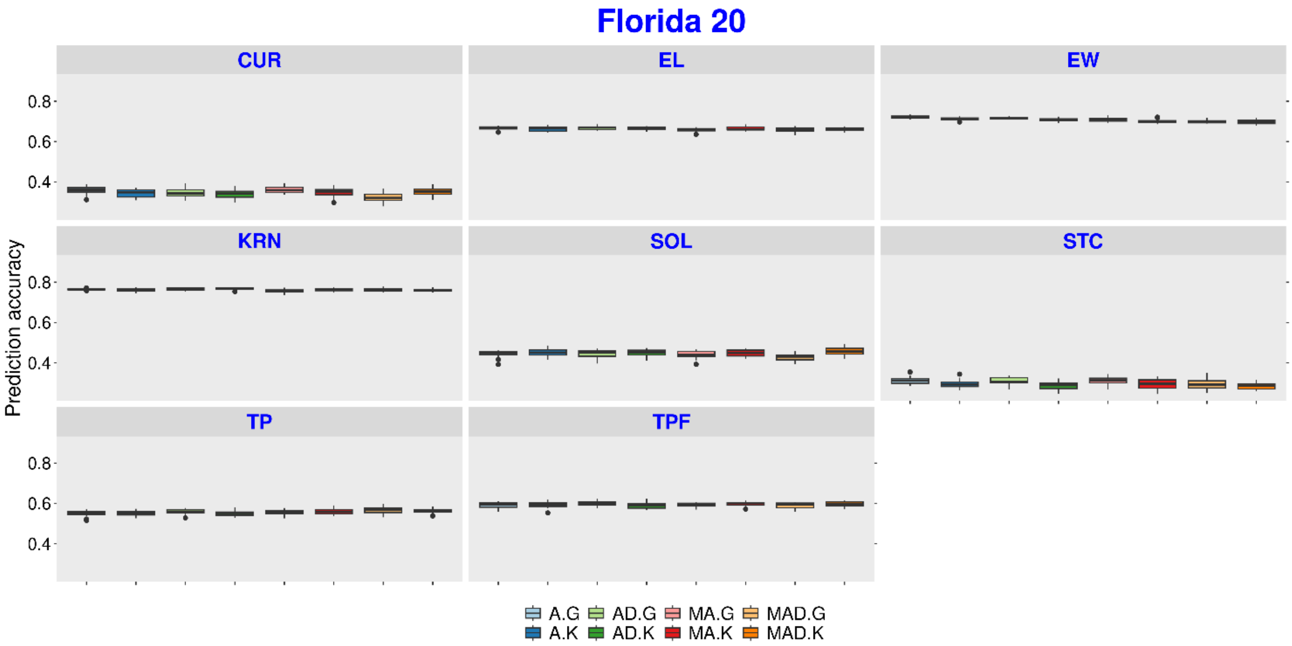

Supplementary Figure S9. Prediction accuracy for eigth traits using eigth four different models under GBLUP and RKHS predictions for Florida 2020 site via CV1 scheme. CUR: curvature. EL: ear length. EW: ear width. KRN: kernel row number. SOL: solidity, STC: stand count. TP: taper. TPF: tip fill. A.G: GBLUP single-trait with additive effect. AD.G: GBLUP single-trait with additive effect and dominance effect. MA.G: GBLUP multi-trait with additive effect. MAD.G: GBLUP multi-trait with additive effect and dominance effect. A.K: RKHS single-trait with additive effect. AD.K: RKHS single-trait with additive effect and dominance effect. MA.K: RKHS multi-trait with additive effect. MAD.K: RKHS multi-trait with additive effect and dominance effect.

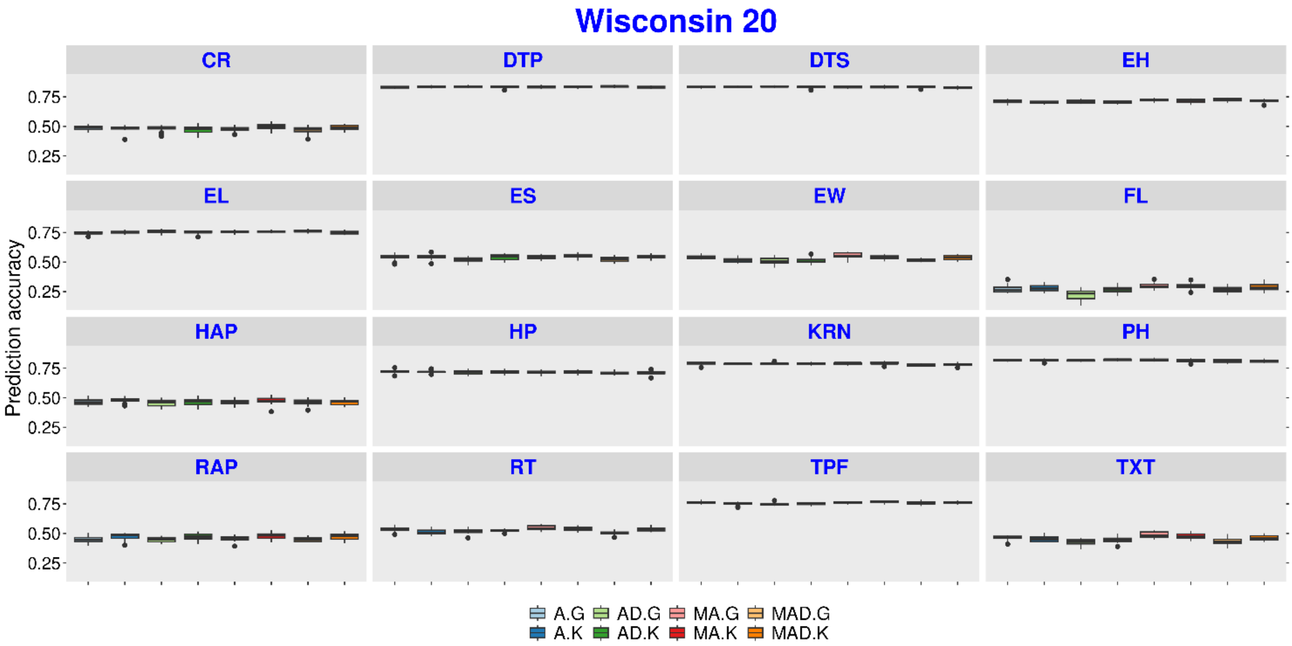

Supplementary Figure S10. Prediction accuracy for eigth traits using eigth four different models under GBLUP and RKHS predictions for Wisconsin 2020 site via CV1 scheme. CR: color rate. DTP: days to pollination. DTS: days to silking. EH: ear height. EL: ear length. ES: ear shape. EW: ear width. FL: flavor. HAP: husk appearance. HP: husk protection. KRN: kernel row number. PH: plant height. RAP: Row appearance. RT: rating. TPF: tip fill. TXT: texture. A.G: GBLUP single-trait with additive effect. AD.G: GBLUP single-trait with additive effect and dominance effect. MA.G: GBLUP multi-trait with additive effect. MAD.G: GBLUP multi-trait with additive effect and dominance effect. A.K: RKHS single-trait with additive effect. AD.K: RKHS single-trait with additive effect and dominance effect. MA.K: RKHS multi-trait with additive effect. MAD.K: RKHS multi-trait with additive effect and dominance effect.
